# Combined mass spectrometry and computational analysis of cyanine dyes and their structural analogues

**DOI:** 10.1039/d5ra07246h

**Published:** 2026-01-28

**Authors:** Irena Đapić, Renata Kobetić, Atanas Kurutos, Tana Tandarić, Ružica Šoić, Robert Vianello

**Affiliations:** a Laboratory for Synthetic Methodologies in Organic Chemistry, Division of Organic Chemistry and Biochemistry, Ruđer Bošković Institute Zagreb Croatia idapic@irb.hr; b Laboratory for Biomolecular Interactions and Spectroscopy, Division of Organic Chemistry and Biochemistry, Ruđer Bošković Institute Zagreb Croatia; c Institute of Organic Chemistry with Centre of Phytochemistry, Bulgarian Academy of Sciences Sofia Bulgaria; d Laboratory for the Computational Design and Synthesis of Functional Materials, Division of Organic Chemistry and Biochemistry, Ruđer Bošković Institute Zagreb Croatia robert.vianello@irb.hr

## Abstract

Cyanine dyes are widely used in biological imaging and labelling, yet the influence of halide counterions on their gas-phase stability and fragmentation remains poorly understood. Here we report a combined high-resolution mass spectrometry and computational study of asymmetric monomethine cyanine dyes bearing one to four positive charges, explicitly elucidating the mechanistic role of halide ions. We demonstrate that iodide uniquely promotes stable gas-phase self-assembly of cyanine dyes, forming higher-order clusters up to pentamers, whereas bromide and chloride do not. Tandem MS reveals pronounced halide-dependent fragmentation energetics, with iodide-containing complexes displaying enhanced stability and distinct product-ion distributions. These iodide-dye anion–π interactions support prior evidence of enhanced dye cell membrane permeability and mitochondrial accumulation. Density functional theory calculations rationalize these observations by identifying an S_N_2-like halide-assisted C–N bond cleavage mechanism, most favorable for iodide, which governs key fragmentation pathways. By integrating MS^*n*^ experiments with computational analysis, this work moves beyond descriptive fragmentation studies and provides a mechanistic framework for halide-mediated behavior of cyanine dyes, with implications for mass spectrometric characterization of cyanine-labelled biomolecules and related imaging probes.

## Abstract 2

Cyanine dyes are widely used in biological imaging and labelling, yet the influence of halide counterions on their gas-phase stability and fragmentation remains poorly understood.

## Graphical abstract

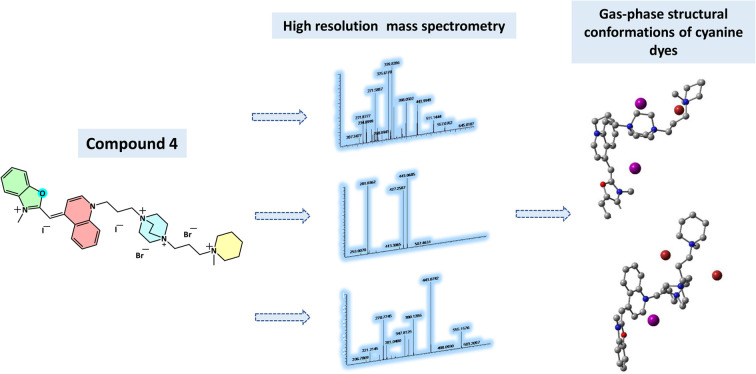

## 1. Introduction

Cyanine dyes have been widely used in biological systems, playing important roles in cellular biology and drug design. However, they are probably best known for their ability to act as fluorophores. Their wide use ranges from detection of tumours, labelling of nucleic acids or selective recognition of DNA and RNA.^1–4^ Matrix assisted laser desorption/ionization mass spectrometry (MALDI-MS) studies described their use for labelling oligosaccharides whereas labelled *N*-glycans from chicken ovalbumin could be detected regardless of the MALDI matrix used.^5^ Cyanine dyes represent an important tool in tumour treatment and recent work reported novel superparamagnetic iron oxide nanoparticles (SPIONs) combined with NIR fluorescent dye indocyanine (cyanine 7, Cy7) to create a dual-modality imaging tool for visualizing glioblastoma (GBM).^6^ Moreover, for prostate cancer treatment, a novel inhibitor combined a nontoxic, fluorescent heptamethine carbocyanine dye with a near-infrared (NIR) emission maxima and a monoamine oxidase A (MAO A) inhibitor to enhance delivery to cancerous lesions.^7^ A novel tumor-targeting NIR dye–MAO A inhibitor conjugate showed high efficiency in targeting tumours and might represent a platform for further development of anti-tumour drugs. Large interest for the use of cyanine dyes is also supported by the studies of their photoinduced dynamics revealing complex photophysics of simple 3,3′-diethylthiacyanine dye^8^ or their careful design to be readily tuned for imaging using intrinsic molecular mechanisms.^9^ A key characteristic of cyanine dyes is their ability to be precisely designed for targeted delivery into specific subcellular locations, such as the mitochondria, nucleus, or Golgi apparatus.^10–12^ This feature enhances their potential for applications in cellular imaging and therapeutic interventions.^13,14^

Despite their extensive use, mass spectrometry studies of cyanine dyes have remained surprisingly limited, particularly with respect to multiply charged systems and counterion effects. Existing MS investigations have largely focused on cataloguing fragmentation pathways of singly charged dyes or on identifying diagnostic ions, often without considering the role of halide counterions beyond simple charge compensation.^15^ As a consequence, the influence of halide identity on gas-phase stability, clustering, and fragmentation energetics of cyanine dyes remains largely unexplored.

Even though the study of anion–π interactions was pioneered by Hiraoka in 1986 focusing on the interaction of hexafluorobenzene and halogens (Cl^−^, Br^−^, and I^−^),^16^ the interactions between anions and aromatic systems have only recently captured significant interest as an important aspect of supramolecular chemistry. Recent studies recognized the functional importance of these weak interactions dominated by electrostatic attractions and ion-induced polarizability.^17^ Electrospray ionization FT-ICR tandem mass spectrometric studies on anion–π complexes between naphthalene diimides and various anions revealed complexes with iodide, dihydrogen phosphate, triflate, and chlorate. Further experiments and computational studies indicated reduced complex stability as the π-acidity of the arene decreased.^18^ In another study, shape-persistent oligonaphthalene diimides were designed that effectively facilitated chloride-selective transport through the membrane *via* multi-ion hopping. End-group design showed to be critical for the function of “anion–π slides” and this method addresses the challenge of tight binding that restricts anion movement, while weak interactions hinder transport, by mimicking nature's strategy of multiple cooperative binding.^19–24^ Recent work demonstrated that the extension of the π-systems of aryl urea-substituted fatty acids by introduction of a second phenyl group enhanced proton transport in vesicle studies, indicating improved charge delocalization by the urea anion binding group to produce membrane-permeable complexes.^25^ While such interactions have been investigated for π-acidic systems and designed receptors, their relevance to polymethine cyanine dyes, particularly under mass spectrometric conditions, has not been systematically addressed. Moreover, the potential role of halide ions as active participants in fragmentation processes, rather than passive spectators, has not been demonstrated for cyanine dyes.

Electrospray ionization (ESI) is most widely used ionization source in mass spectrometry as it is declared as “soft” ionization technique that produces minimal fragmentation during the ionization process. However, there are still technical challenges existing such as in-source fragmentation (ISF) requiring need for optimal ISF settings since unintended ISF can cause low confidence in the identity confirmation process, and misannotation of peaks.^26^ Overall, having it improved can help in identification, particularly in the case of small molecules and metabolites.^27,28^ Fragmentor voltage (FV) or declustering potential is one of the vital elements in ion source settings and selected FV significantly affects the abundance of the primary analyte ion and the level of in-source fragmentation, enabling the optimal selection of qualifier fragments for analytes. Previous studies showed important function of FV in quantitative analyses of hormones^29^ and metabolite annotation, indicating that FV has key influence on spectra quality.^30,31^

**Fig. 1:**
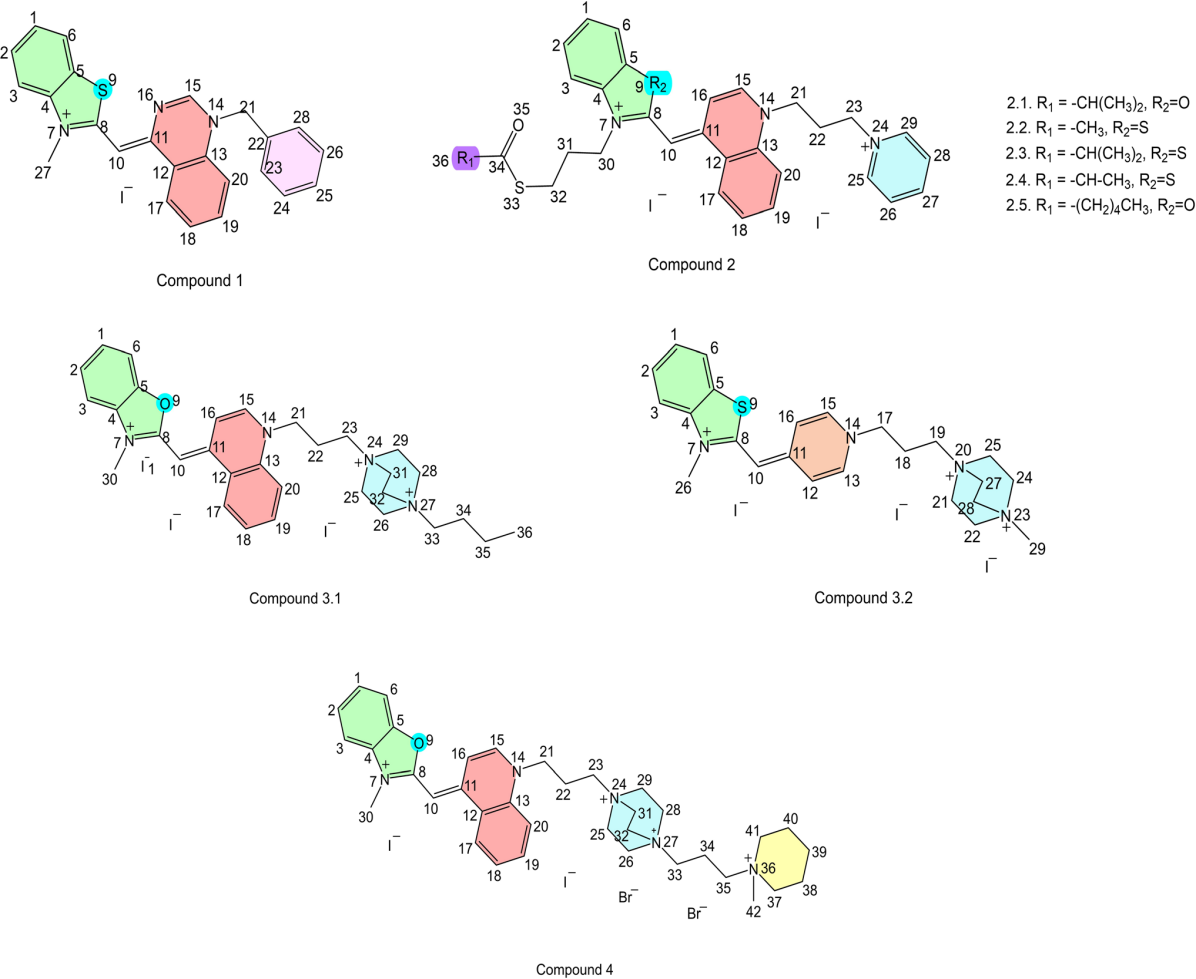
Structures of the asymmetric monomethine cyanine dyes 1–4 investigated in this study. The series comprises mono-, di-, tri-, and tetra-cationic dyes differing in charge state and counterion identity, enabling systematic evaluation of halide-dependent gas-phase behavior.

Here, we present the first integrated experimental and computational study that explicitly links halide identity to gas-phase clustering, fragmentation energetics, and reaction mechanisms in cyanine dyes across multiple charge states (Fig. 1). Using a series of asymmetric monomethine cyanine dyes bearing one to four positive charges, we demonstrate that iodide uniquely promotes the formation of stable, higher-order gas-phase clusters, in contrast to bromide and chloride. High-resolution MS^*n*^ experiments reveal pronounced halide-dependent fragmentation pathways, which are rationalized by density functional theory calculations showing S_N_2-like halide-assisted C–N bond cleavage, most favorable for iodide. Investigated dyes have previously been studied in our group for their biological activity and showed potential to bind different polynucleotide secondary structure motifs.^23^

By combining high-resolution tandem mass spectrometry, systematic counterion variation, and computational analysis, this work goes beyond descriptive fragmentation studies and provides a mechanistic framework for understanding halide-mediated behavior of cyanine dyes in the gas phase. These insights are directly relevant for interpreting MS data of cyanine-labeled biomolecules and offer a molecular basis for the distinct biological and physicochemical properties associated with iodide-containing cyanine dyes.

## 2. Experimental section

### 2.1. Preparation of cyanine dyes

Lipophilic cyanine dyes with several cationic quaternary ammonium moieties (Fig. 1) were prepared according to previously described method.^32^ Strategic modifications were studied to provide insights into how different charged groups affect the performance and behavior of cyanine dyes in MS. To investigate for structural variations DABCO was introduced into the structure for compounds 2–4. Additionally, to further examine the effects of charge on the compounds, DABCO underwent quaternization with alkyl chain (compound 3) or with piperidine cation (compound 4).

### 2.2. Mass spectrometry measurements

Structural characterization and non-covalent interactions of compounds presented at Fig. 1 were studied by ESI, using high resolution mass spectrometry for MS^*n*^ studies, and triple-quadrupole ESI-MS/MS for clusters characterization. The fragmentation pathways for all analyzed compounds were proposed based on high resolution MS^*n*^ spectra of positive ions [M–I^−^]^+^ or negative ions [M + I^−^]^−^ as well as iodide adducts.

### 2.3. Structural characterization of cyanine dyes with high resolution mass spectrometry

Tandem high resolution mass spectrometry ESI-MS^*n*^ experiments were performed on LTQ XL Orbitrap mass spectrometer (Thermo Scientific MA, USA). Data were collected on ITMS ESI positive or negative polarity mode with the following parameters: isolation window width was set to ±1 Da, ion source capillary temperature 275 °C, spray voltage 5 kV, capillary voltage 50 V. Nitrogen was used as sheath gas with flow rate of 15 arb, auxiliary gas with flow rate 5 arb and sweep gas 0.02 arb. Helium was used as collision gas and spectra were recorded in range of 50–2000*m*/*z*. The samples were prepared in mixture water : methanol (1 : 1) at a concentration of 1 mg mL^−1^ and injected into the ESI source of the mass spectrometer by a syringe pump at a flow rate of 1 µL min^−1^.

### 2.4. Mass spectrometry for identification of clusters

To characterize formation of clusters for compound 2 data were acquired using an Agilent 6420 Triple Quad mass spectrometer equipped with an ESI interface operated in the positive and negative modes (Agilent Technologies, Palo Alto, CA, USA). The samples were prepared in water, methanol, acetonitrile, water/methanol, and water/acetonitrile with and without NaCl or NH_4_OAc to a concentration of 0.05 mg mL^−1^ and directly injected. The infusion into the mass spectrometer was performed at a flow rate of 3 µL min^−1^. Nitrogen was used as an auxiliary and sheath gas. The spray voltage was set at 4.5 kV. The capillary temperature was 150–300 °C, and the voltage range of the collision cell was 50–200 V. The full mass spectra were acquired over the mass range 10–2350*m*/*z*. Mass Hunter software (Agilent Technologies, Inc. 2006–2007) was used for data acquisition and analysis and isolation window width was set to ±1 Da to perform MS/MS experiments.

### 2.5. Computational analysis

Computational analysis was focused of systems 2.1 and 4 as representative cases and investigated their 1 : 1 Cl^−^ and I^−^ complexes to identify their intrinsic tendencies for these anions. To probe the conformational flexibility of individual dyes and their halide complexes, we initially employed the Conformer-Rotamer Ensemble Sampling Tool (CREST) analysis.^28^ CREST calculations use the GFN2-xTB tight binding Hamiltonian,^29^ the generalized Born with surface area contributions (GBSA) continuum model for water solvent,^30^ and the iMTD-GC metadynamics-based exploration of conformational space for the collective variables.^31^ Ten most representative structures obtained through the CREST analysis were then reoptimized in the Gaussian 16 software^32^ employing either the M06-2X/LANL2DZdp model with ECP potentials for sulfur, chlorine and iodine for the gas-phase calculations or the same approach supplemented with the implicit SMD solvation corresponding to the aqueous solution. This offered most stable structures in both phases, and their geometries and energies were used in obtaining thermodynamic and kinetic values discussed throughout the text. Such a DFT setup was selected following literature recommendations for the considered properties and type of systems.^33^ Each geometry optimization was followed by the vibrational frequency analysis, offering thermal corrections so that all presented results correspond to Gibbs free energies at room temperature and normal pressure. All transition state structures were located through the scan procedure, employing both 1D and 2D scans, the latter specifically utilized to exclude the possibility for concerted mechanisms, and then fully optimized as saddle points on the potential energy surface. Apart from the visualization of the obtained negative frequencies, the validity of all transition states was confirmed through IRC calculations in both directions.

## 3. Results and discussion

The Results and discussion section is structured to highlight three central advances of this work: (i) the discovery of iodide-driven gas-phase self-assembly of cyanine dyes into higher-order clusters, (ii) the demonstration that halide identity governs fragmentation energetics and product ion stability, and (iii) the identification of an S_N_2-like halide-assisted mechanism for C–N bond cleavage, supported by both MS^*n*^ experiments and DFT calculations. Given the close structural similarity of the investigated cyanine dyes and the highly analogous fragmentation behavior observed across compounds differing primarily in charge state or counterion identity, the MS^*n*^ discussion below focuses on representative examples. Detailed fragmentation schemes and complete MS^*n*^ datasets for all remaining compounds are provided in the SI (Fig. S1–S10).

### 3.1. Assessment of in-source fragmentation voltage for cyanine dyes

To evaluate the influence of fragmentor voltage (FV) on ion stability and in-source fragmentation (ISF), extracted ion chromatograms (EICs) were analyzed for representative dicationic and tricationic cyanine dyes. Compound 3.2 was selected as a representative system due to its well-defined charge states and reproducible ion response.

**Fig. 2:**
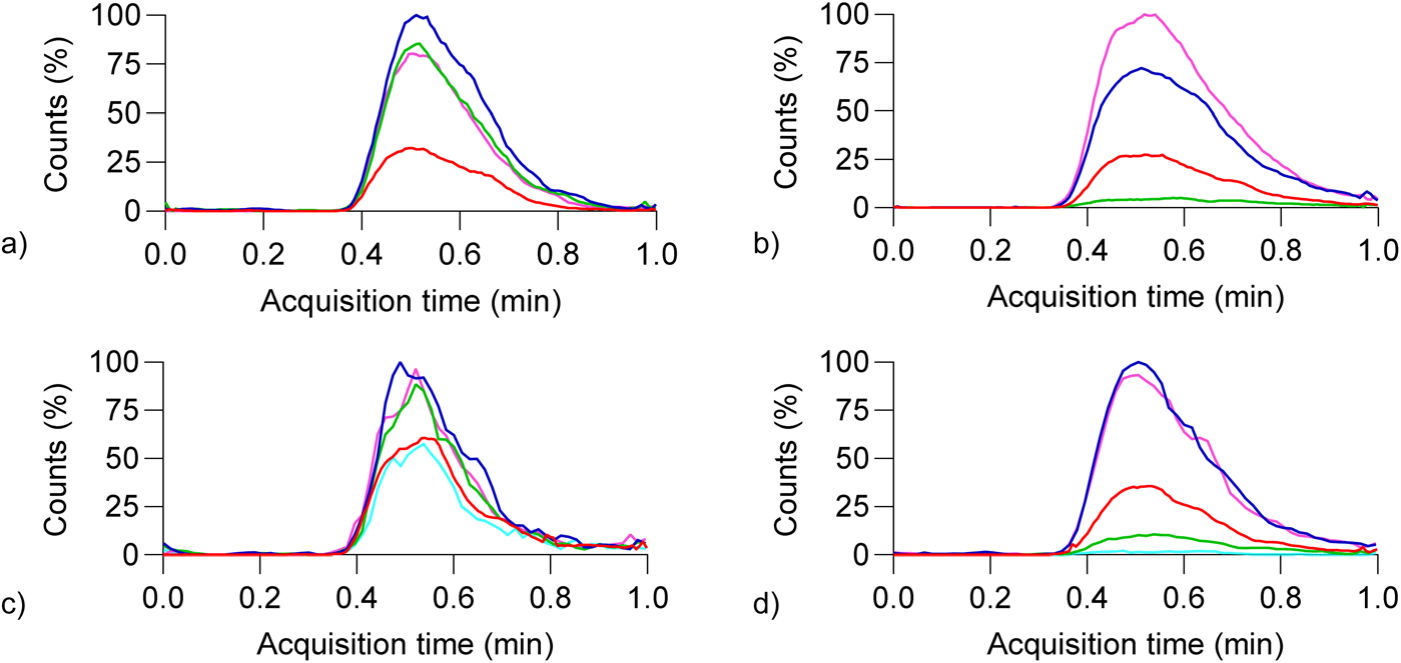
Effect of fragmentor voltage (FV) on in-source fragmentation and charge-state distribution for representative tricationic molecules 3.2 (a and b) and 3.1 (c and d) dissolved in 1 : 1 water : methanol (*c* = 1 mg mL^−1^), 10 V (red), 50 V (pink), 100 V (blue), 150 V (green), and 200 V (light blue). (a) *m*/*z* 662.8; (b) *m*/*z* 268; (c) *m*/*z* 739; (d) *m*/*z* 306. Data collected on Agilent 6420 Triple Quad mass spectrometer.

**Fig. 3:**
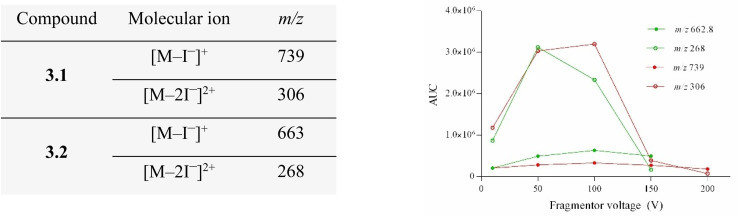
Fragmentor-voltage-dependent ion abundance and area-under-the-curve analysis for representative charge states of 3.1 (red) and 3.2 (green) across various FV values. Single charged ions at *m*/*z* 739 (3.1) and *m*/*z* 662.9 (3.2) are shown with filled circle, while double charged ions at *m*/*z* 306 (3.1) and *m*/*z* 268 (3.2) with empty circles. Data acquisition was performed on Agilent 6420 Triple Quad mass spectrometer. Samples were dissolved in 1 : 1 water : methanol (*c* = 1 mg mL^−1^).

Increasing FV resulted in progressively richer total ion current (TIC) spectra (Fig. S1), indicating enhanced ISF of molecular ions. For compound 3.2, EICs at *m*/*z* 662.8 ([M–I^−^]^+^) and *m*/*z* 268 ([M–2I^−^]^2+^) revealed a critical FV range between 100 and 150 V. Beyond this range, ion abundance decreased, consistent with excessive fragmentation. At lower FV values, doubly charged ions were more abundant than singly charged species, likely reflecting enhanced stability of multiply charged ions after counterion loss (Fig. 2 and 3).

We compared the area under the curve (AUC) of the EIC for compounds 3.1 and 3.2 at different FV values, focusing on their molecular ions following the loss of one and two iodide ions. Interestingly, data show that for FV at 50 and 100 V ions after the loss of two iodides have higher AUC as compared to their corresponding molecular ion after loss of one iodide ion. This might be attributed to the fact that the molecule is more stable after loss of one iodide. However, upon increasing the FV to 150, the *m*/*z* 268 value for 3.2 appears slightly lower compared to *m*/*z* 662.9, whereas for 3.1, both ions exhibit similar values. After further increasing FV to 200 for 3.1, values of *m*/*z* 306 decreases and shows less abundance as compared to *m*/*z* 739. This might indicate that very high FV has a strong fragmentation effect and we no longer observe molecular ions, but rather more rich TIC spectra (Fig. S2).

### 3.2. High resolution mass spectrometry for structural characterization of cyanine dyes

To elucidate fragmentation pathways across different charge states, high-resolution MS^*n*^ experiments were performed on representative singly charged and multiply charged cyanine dyes. Compound 1 was selected as a representative singly charged dye, while compound 4 was chosen to represent quaternary-charged systems.

#### 3.2.1. Representative singly charged dye (compound 1)

**Fig. 4:**
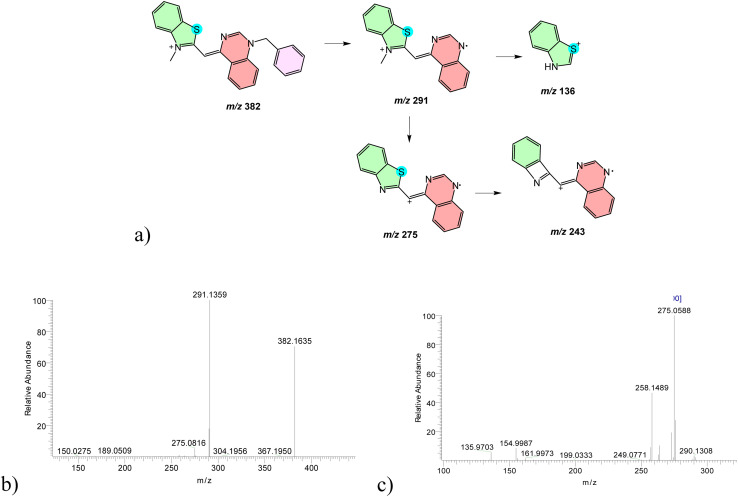
Representative MS^*n*^ fragmentation pathways of a singly charged cyanine dye 1. High-resolution MS^2^–MS^3^ spectra establish baseline fragmentation behavior, including heterocycle cleavage and sequential alkyl loss, serving as a reference for higher charge states. (a) Annotated molecular structures and fragmentation schemes of parent ion at *m*/*z* 382 assigned as [M–I^−^]^+^; (b) MS^2^ spectra of *m*/*z* 382 at CID 25 eV; (c) MS^3^ spectra of *m*/*z* 382 at CID 35 eV → 291 at CID 45 eV.

In positive ion mode, compound 1 displayed a dominant molecular ion at *m*/*z* 382.1667, corresponding to [M–I^−^]^+^ following iodide loss (Fig. S3a). Low-energy CID produced limited fragmentation, whereas increased CID energy led to well-defined cleavage pathways. The principal fragmentation involved homolytic cleavage between the N14–C21 bond, generating a product ion at *m*/*z* 291.1359, followed by sequential loss of methyl and heterocyclic fragments (Fig. 4). MS^3^ and MS^4^ experiments further confirmed benzothiazole ring cleavage and sulfur elimination pathways (Fig. 4c and S3d). Comparable fragmentation behavior was observed using triple-quadrupole MS, confirming the robustness of the proposed pathways across platforms (Fig. S4).

#### 3.2.2. Representative quaternary-charged dye (compound 4)

**Scheme 1:**
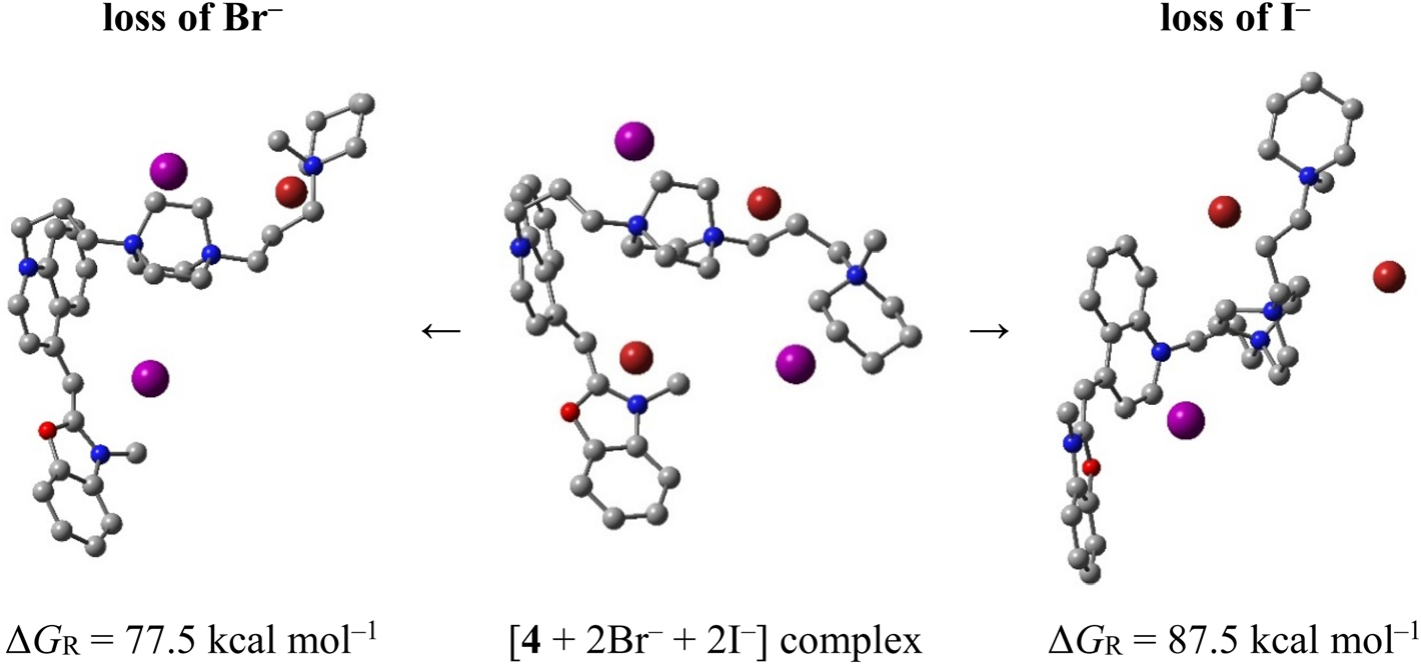
Computed reaction Gibbs free energy change following halide loss from the complex of 4 with two Br^−^ (in pink) and two I^−^ (in red), M06-2X/LANL2DZdp results.

Having in mind advantages of quaternary charged systems, such as better solubility, solution stability, and improved nucleic acids affinity, we further examined the molecular structure of 4. It exhibited multiple charge states resulting from partial halide loss, including ions at *m*/*z* 411.6667 ([M–2Br^−^]^2+^), *m*/*z* 388.6667 ([M–Br^−^–I^−^]^2+^), and *m*/*z* 232.1667 ([M–2Br^−^–I^−^]^3+^) (Fig. S5a). The isotopic pattern of *m*/*z* 388 confirmed retention of a single bromide. CID of *m*/*z* 388 revealed preferential bromide loss accompanied by alkyl cleavage and methyl loss from tertiary nitrogens, showing a higher affinity of the dye for iodide over bromide. This is supported by DFT data confirming that 4 liberates Br^−^ much easier than I^−^. Specifically, the Gibbs free energy required to strip Br^−^ from the complex involving 4 with two iodide and two bromide is 77.5 kcal mol^−1^, which is significantly more endergonic at 87.5 kcal mol^−1^ for the analogous I^−^ liberation (Scheme 1).

**Fig. 5:**
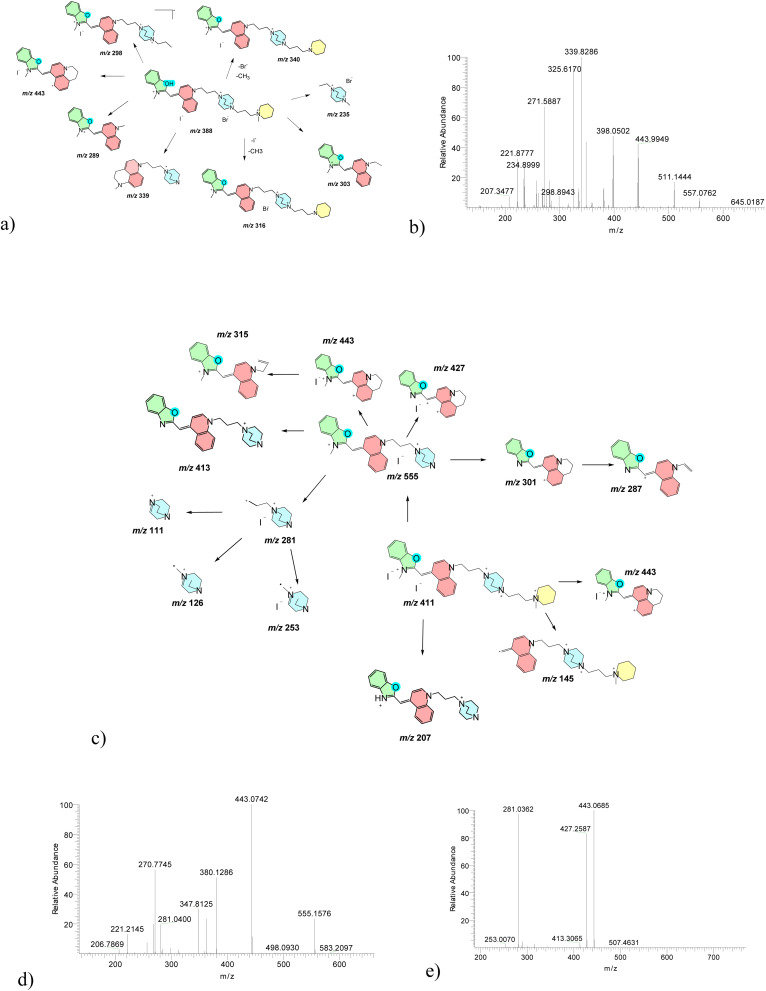
Halide-dependent fragmentation of a quaternary-charged cyanine dye 4. CID spectra reveal preferential bromide loss and retention of iodide in high-*m*/*z* fragments, highlighting the distinct gas-phase behavior of different halide counterions. (a) Annotated molecular structures and fragmentation scheme of parent ion at *m*/*z* 388, [M–I^−^–Br^−^]^2+^; (b) MS^2^ spectra of *m*/*z* 388 at CID 30 eV; (c) annotated molecular structures and fragmentation scheme of parent ion at *m*/*z* 411, [M–2Br^−^]^2+^; (d) MS^2^ spectra of *m*/*z* 411 at CID 30 eV, (e) MS^3^ spectra of *m*/*z* 411 at CID 20 eV → 555 at CID 30 eV.

MS^2^ spectra of *m*/*z* 903 (Fig. S5b) shows fragment at *m*/*z* 762 that might be attributed to a loss of I^−^ and a methyl group. MS^2^ spectra of parent ion (M–Br–I)^2+^ (*m*/*z* 388) showed peak at *m*/*z* 340, belonging to (M–2Br^−^–I^−^)^3+^, and the piperidine methyl loss. Low intensity analogue cation at *m*/*z* 316 was observed for (M–Br–2I)^2+^ and the piperidine methyl loss (Fig. 5b). The latter is consistent with DFT data showing the piperidine *N*-methyl removal in a heterolytic fashion as a methyl cation (–CH_3_^+^) is by −23.1 kcal mol^−1^ more favorable than the analogous process at the benzoxazole nitrogen.

Fragmentation of *m*/*z* 411 revealed formation of iodide-retaining fragments coupled to either the benzene π-system or DABCO (Fig. 5). Subsequent MS^3^ and MS^4^ experiments showed sequential alkyl cleavage, ring opening, and heterolytic C–N bond dissociation pathways, with iodide retained in several high-*m*/*z* fragments.

Tricationic 3.1 and 3.2 displayed fragmentation patterns closely analogous to those observed for 4, including halide retention, alkyl-chain cleavage adjacent to quaternary nitrogen centers, and formation of iodide-stabilized cations (Fig. S10). As these pathways are mechanistically redundant, only representative spectra are discussed here, while complete MS^*n*^ datasets are included in the SI.

### 3.3. Intermolecular interactions and cluster formation in dicationic cyanine dyes

Gas-phase cluster formation was investigated for dicationic cyanine dyes 2.1–2.5 to assess the role of halide counterions in mediating intermolecular interactions. Compound 2.1 is discussed as a representative system, as all examined dyes exhibited qualitatively identical clustering behavior.

**Fig. 6:**
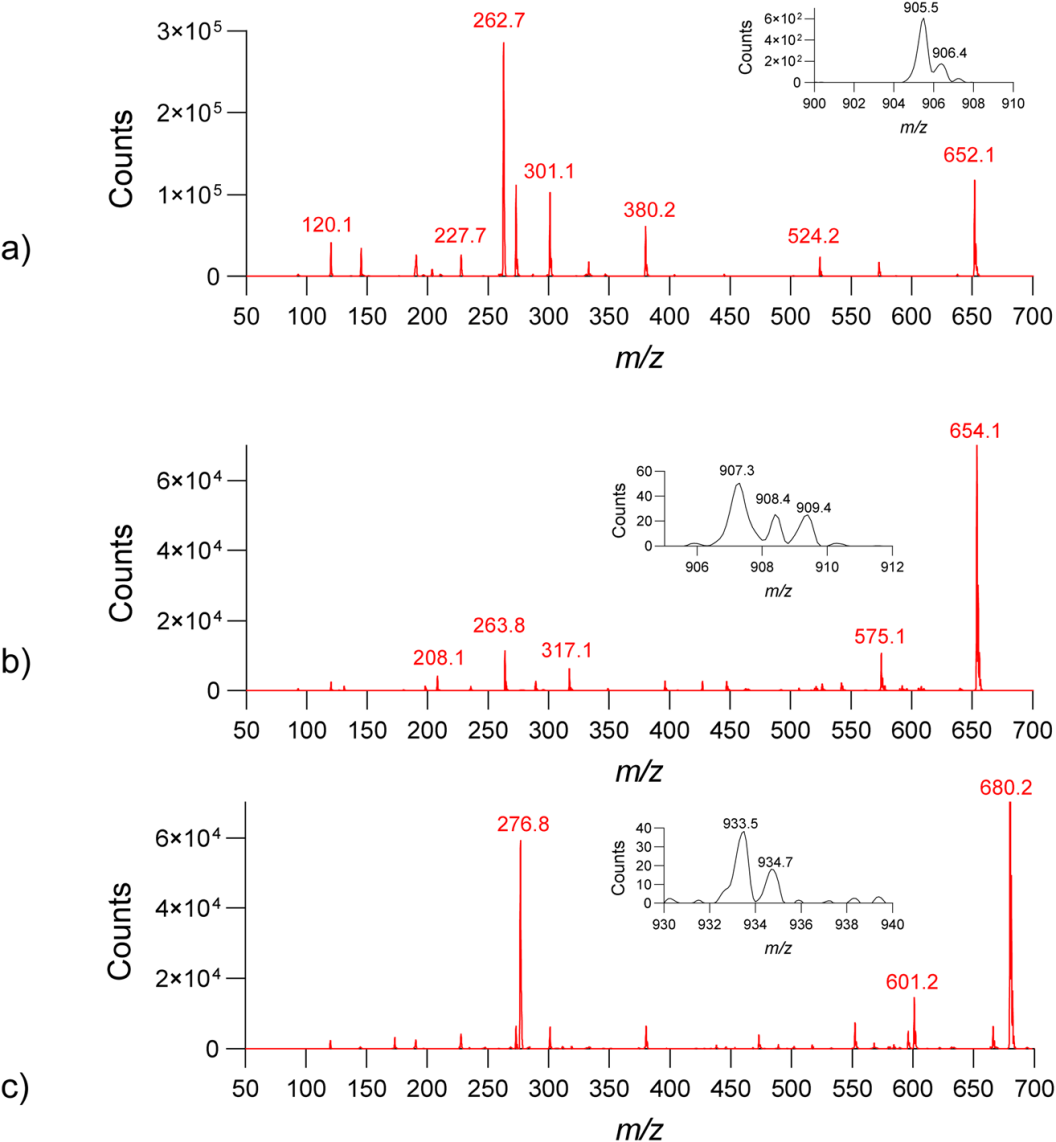
Observation of iodide-mediated gas-phase cluster formation for a representative dicationic cyanine dyes 2.1, 2.4, and 2.5 in ESI^+^ and ESI^−^ (see insets). MS ESI^+^ spectra of 2.1 (a), 2.4 (b), and 2.5 (c). Iodide complexes observed in the negative mode with signals at *m*/*z* 905, 907, and 933 assigned as [M + I^−^]^−^, are shown as insets. The single and double-charged species [M–I^−^]^+^ and [M–2I^−^]^2+^ were also observed.

**Fig. 7:**
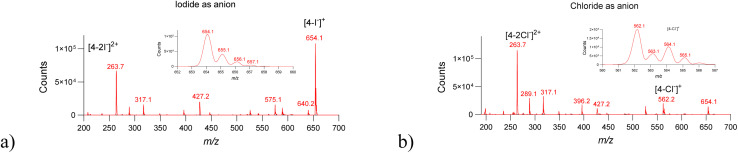
Comparison of iodide- (a) and chloride-containing (b) cyanine dye 4 reveals counterion-specific clustering behavior. While iodide promotes higher-order cluster formation, chloride-containing analogues show only monomeric species, underscoring the unique role of iodide. Full scan ESI^+^ MS for 2.4 dissolved in water (*c* = 10^−5^ mol L^−1^). Insert shows the signals of the singly charged species containing chloride or iodide. Double-charged ions had a higher abundance when chloride was counter-ion at the same cone voltage value (135 V).

In ESI positive mode, 2.1 produced singly and doubly charged species at *m*/*z* 652.2 ([M–I^−^]^+^) and *m*/*z* 262.7 ([M–2I^−^]^2+^), respectively (Fig. 6). In addition, higher-order clusters involving multiple dye molecules and I^−^ ions were observed, up to pentamers (Fig. S8). These clusters persisted across a wide range of solvents and cone voltages, indicating substantial intrinsic stability. Notably, self-assembled clusters were observed exclusively for iodide-containing dyes. Replacement of iodide with chloride resulted in markedly reduced abundance of halide-containing species and complete suppression of higher-order cluster formation (Fig. 7). MS/MS comparison of iodide and chloride adducts further revealed distinct fragmentation pathways, with iodide complexes exhibiting lower-energy fragmentation and enhanced retention of the halide in high-*m*/*z* fragments (Fig. S9).

Systematic solvent screening and cone-voltage variation confirmed that the observed iodide-mediated clusters are not artefacts of electrospray aggregation but instead reflect specific anion–π interactions intrinsic to the dye structures. Comparable results for compounds 2.4 and 2.5 are provided in Fig. S6 and S7.

**Fig. 8:**
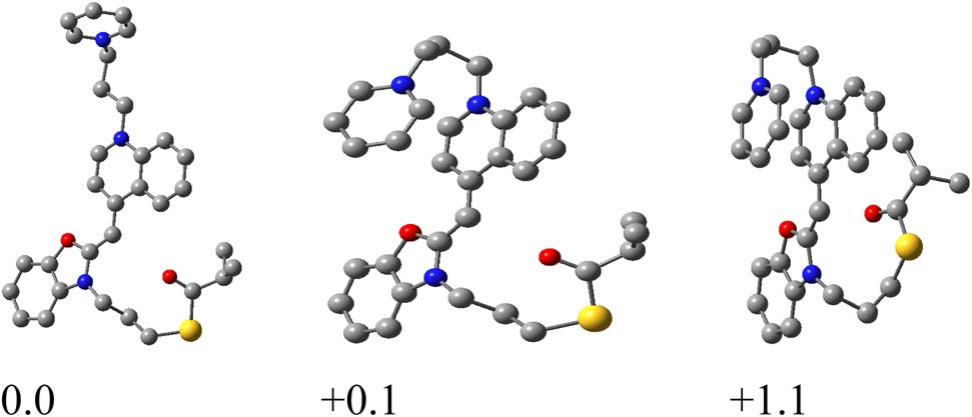
Relevant gas-phase conformations of 2.1 and their relative stabilities (in kcal mol^−1^) as obtained by the M06-2X/LANL2DZdp model. Hydrogen atoms are omitted for clarity.

**Fig. 9:**
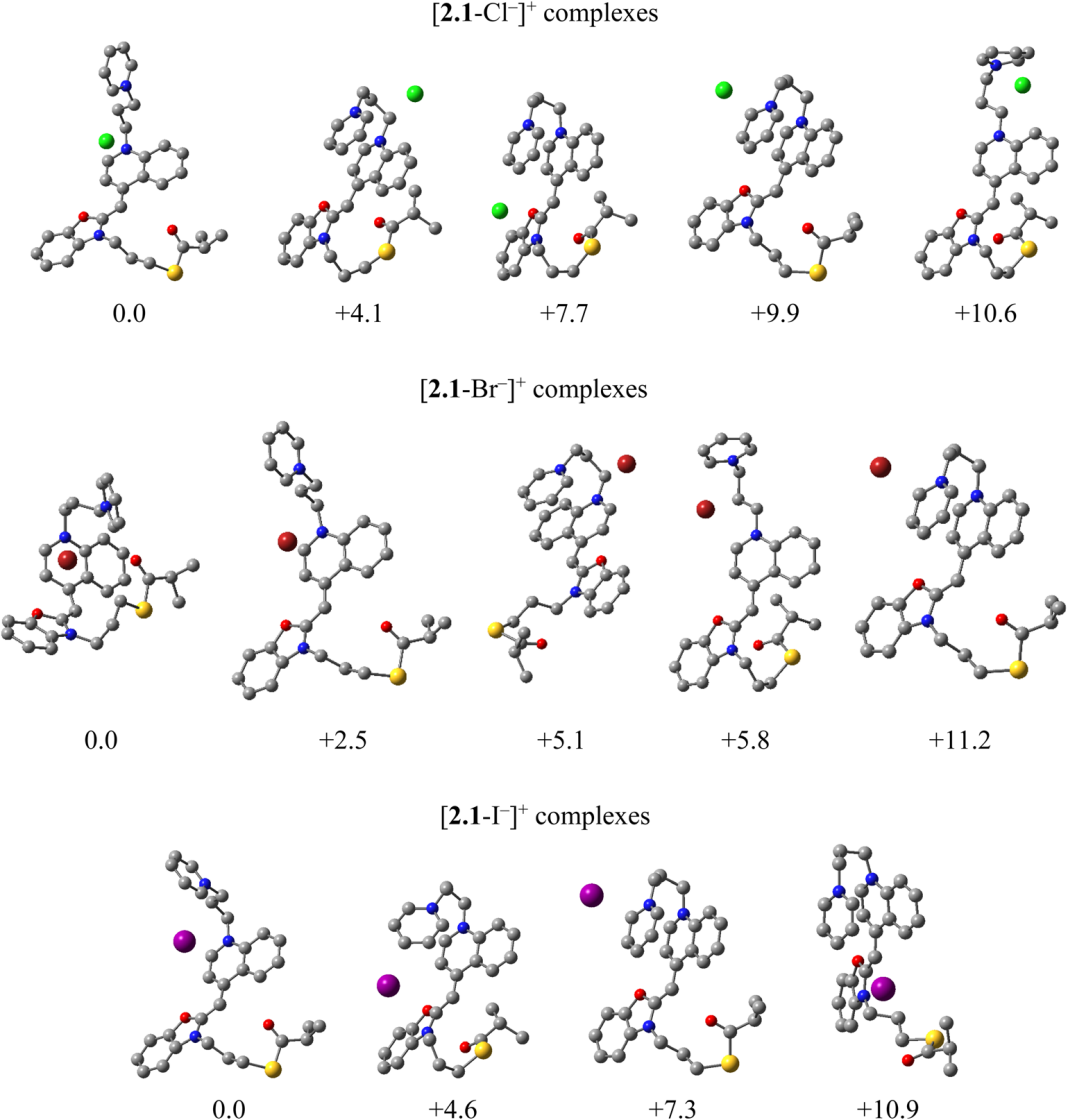
Relative gas-phase stability of 2.1–halide complexes at the M06-2X/LANL2DZdp model following the CREST analysis (in kcal mol^−1^). Hydrogen atoms are omitted for clarity.

To clarify the experimental trends observed in Fig. 7–9, we observe several consistent features across all studied dyes. First, iodide-containing species form stable gas-phase clusters of higher order (up to pentamers), while equivalent chloride analogues do not show such self-assembly. Second, fragmentation pathways differ markedly depending on the counterion: iodide adducts consistently display lower-energy fragmentation, earlier onset of pyridine cleavage, and retention of iodide in several high-*m*/*z* fragments. In contrast, chloride adducts show a reduced abundance of singly charged halide-containing fragments and different bond-cleavage preferences. These systematic differences indicate that halide identity strongly influences both cluster stability and fragmentation behavior, suggesting distinct underlying binding geometries and interaction strengths. To rationalize these experimentally observed halogen-dependent behaviors, we next performed a detailed computational analysis, presented later in the text.

We note that the interpretation of gas-phase cluster formation requires caution, as electrospray ionization can in some cases generate non-specific aggregates that do not correspond to solution-phase assemblies. To address this, we performed a systematic cone-voltage (50–200 V) and solvent screening (water, methanol, acetonitrile, ammonium acetate, NaCl solutions). The observed cluster stoichiometries for iodide-containing species remained invariant across all conditions, and the higher-order clusters (dimers–pentamers) persisted even at elevated cone voltages. Such stability is inconsistent with accidental gas-phase aggregation and is characteristic of specific anion–π-mediated interactions. In contrast, chloride analogues did not form similar clusters under any conditions, further supporting that the observed assemblies reflect intrinsic halide–dye interactions rather than experimental artifacts.

Additionally, tricationic dye 3.1 was examined as a representative benzoxazole-containing system to further evaluate the influence of increased charge density on fragmentation behavior. TIC spectra revealed molecular ions corresponding to the loss of one, two, and three iodide anions (*m*/*z* 739, 306, and 161, respectively; Fig. S10). CID of *m*/*z* 739 generated major fragments at *m*/*z* 555, 480, 443, and 288, consistent with alkyl-chain cleavage, heterolytic C–N bond dissociation, and iodide-stabilized carbocation formation. Importantly, even at elevated collision energies, low-*m*/*z* fragments were largely absent, indicating exceptional stability of iodide-containing complexes in the gas phase.

Fragmentation pathways for compound 3.2 closely mirrored those of 3.1 and compound 4, confirming that halide-dependent behavior is governed primarily by charge density and anion identity rather than subtle structural differences. Detailed fragmentation schemes for all tricationic dyes are provided in the SI.

### 3.4. Computational analysis

Density functional theory (DFT) calculations were performed to rationalize the experimentally observed halide-dependent trends in gas-phase stability, cluster formation, and fragmentation behavior. In particular, the calculations were aimed at explaining (i) the unique ability of iodide to promote stable gas-phase self-assembly, (ii) the pronounced dependence of fragmentation energetics on halide identity, and (iii) the experimentally inferred halide-assisted C–N bond cleavage pathways. By identifying preferred halogen binding geometries, relative binding energies, and activation barriers for key C–N bond cleavages, the computational results provide a mechanistic framework that rationalizes the experimental fragmentation patterns and the unique stabilizing effect of iodide. Compound 2.1 was selected as a representative system, as all studied dyes exhibited qualitatively similar behavior. The results obtained for this system are therefore discussed as illustrative of the broader dye series.

For that purpose, we have first conducted the CREST conformational search that allowed us to elucidate the representative structures of isolated ligands and their halide complexes. These were, then, reoptimized with the DFT M06-2X/LANL2DZdp methodology in both gas-phase and implicit SMD water solution, and discussed throughout the text. Although the M06-2X functional combined with LANL2DZdp pseudopotentials is among the recommended approaches for modeling non-covalent interactions, limitations remain when describing highly polarizable halide–π interactions. Dispersion and charge-transfer contributions involving iodide could be underestimated, and therefore the absolute binding energies should be interpreted with caution. Our computational analysis is therefore intended to rationalize observed MS fragmentation pathways and provide reliable relative trends, rather than exact quantitative predictions.

#### 3.4.1. Halide binding preferences in solution

Molecule 2.1 consists of a rather rigid central core and two very flexible ends, involving pyridine and thioester moieties. Their flexibility is afforded by two propyl linkers connecting them to different sides of the molecular skeleton. This leads to three types of conformations identified in the water solution, which differ in their tendency to undergo the π–π stacking interactions (Fig. S11). Conformational sampling revealed that isolated 2.1 predominantly adopts an “S-shaped” geometry, stabilized by intramolecular π–π interactions between the aromatic units. This conformation is strongly favored over extended geometries, indicating limited structural heterogeneity in solution.

When halide ions are concerned, our results demonstrate their ability to bind to 2.1, generally without disturbing its intrinsically favorable “S-shaped” orientation (Fig. S12). As a common feature, all three halides associate near the cationic pyridine moiety; however, their binding geometries differ markedly. Namely, Cl^−^ and Br^−^ favor binding between adjacent aromatic units, whereas I^−^ preferentially positions above the pyridine ring, with the I⋯N distance of 3.65 Å, the latter closely matching those in crystal structure with analogous structural elements.^17^ We also noticed the type of the structure that can be described as a “sandwich” complex, where halides are positioned between the pyridine and the central aromatic fragments. However, since this disrupts intrinsically favorable π–π stacking contacts between two aromatics, their stabilities are between 2–6 kcal mol^−1^ lower. In addition, the high tendency of I^−^ to engage in anion–π stacking interactions is also reflected in a degeneracy of conformations involving pyridine and benzoxazole stacking (Fig. S12).

Considering the most stable solution structures, the computed binding free energies are −6.3 kcal mol^−1^ for Cl^−^, −3.4 kcal mol^−1^ for Br^−^, and +1.0 kcal mol^−1^ for I^−^. A decreased affinity for I^−^ agrees with a range of experimental observations and is explained by the increased size and polarizability of the anion.^17^ The obtained results clearly point to a significant thermodynamic stability of [2.1–Cl^−^]^+^ and [2.1–Br^−^]^+^ complexes and their predominance in solution, and justify their presence in the MS measurements. Although iodide binding is thermodynamically weaker in solution than chloride or bromide, the computed binding energy of [2.1–I^−^]^+^ indicates that iodide-bound species are accessible (around 15% solution population), consistent with their detection in ESI-MS. These solution-phase results indicate that iodide association is structurally distinct rather than dominant, suggesting that gas-phase effects play a decisive role in the experimentally observed iodide-specific behavior.

#### 3.4.2. Gas-phase halide complexes and fragmentation pathways

The relevant conformations of isolated 2.1 and its halide complexes, identified in the previous stage, were reconsidered in the gas-phase and further analyzed for the most preferred fragmentation pathways, particularly focusing on pyridine liberation experimentally determined as significant.

In the gas phase, the preferred dye conformations become more extended, and intramolecular π–π stacking is significantly reduced (Fig. 8). This structural reorganization strongly influences halide binding geometry and subsequent fragmentation pathways.

**Scheme 2:**
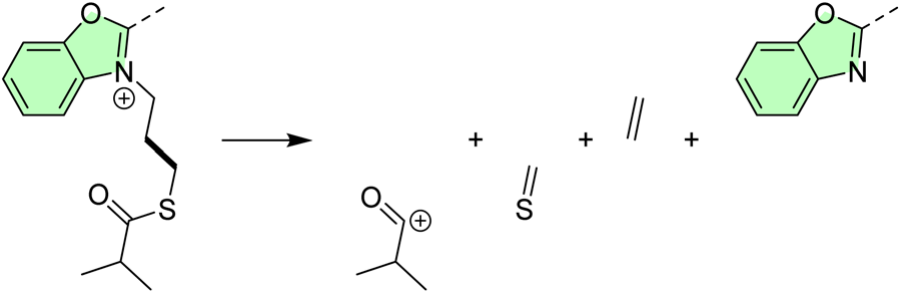
Rearrangement reactions following the heterolytic C10–C11 cleavage.

For isolated 2.1, the majority of the inspected C–C or C–heteroatom bonds most preferably cleave in the homolytic fashion (Table 1), leaving radical centers on both ends. Their detection in MS spectra then relies on chemically induced positive charges within the pyridine and benzoxazole units, or on subsequent rearrangement processes. The computed homolytic bond dissociation energies stretch between around 20 and 80 kcal mol^−1^ in all instances, except for the central C6–C7 bond where it is increased to almost 142 kcal mol^−1^. The latter is due to its partial double bond character as a result of the electronic resonance. Still, in several cases, the heterolytic cleavage becomes important and thermodynamically comparable, or even surpassing the analogous homolytic processes. This holds especially in situations where the primary cleavage is followed by a secondary fragmentation that stabilizes the product state, or in cases where the excess anionic charge forms a stable aromatic structure and liberates it as a neutral fragment. In this context, it is worth mentioning the C10–C11 cleavage that is accompanied by the liberation of the neutral ethylene (C_2_H_4_) in the anionic part and the neutral H_2_C=S in the cationic part (Scheme 2). This gives the heterolytic bond energy of 71.7 kcal mol^−1^, being 9.0 kcal mol^−1^ more favorable than the analogous homolytic cleavage.

**Table 1:** Computed bond dissociation energies (BDE) in 2.1 together with their mechanistic description. All values are in kcal mol^−1^ and are obtained by the M06-2X/LANL2DZdp model

Structure	Bond	BDE	Cleavage mechanism
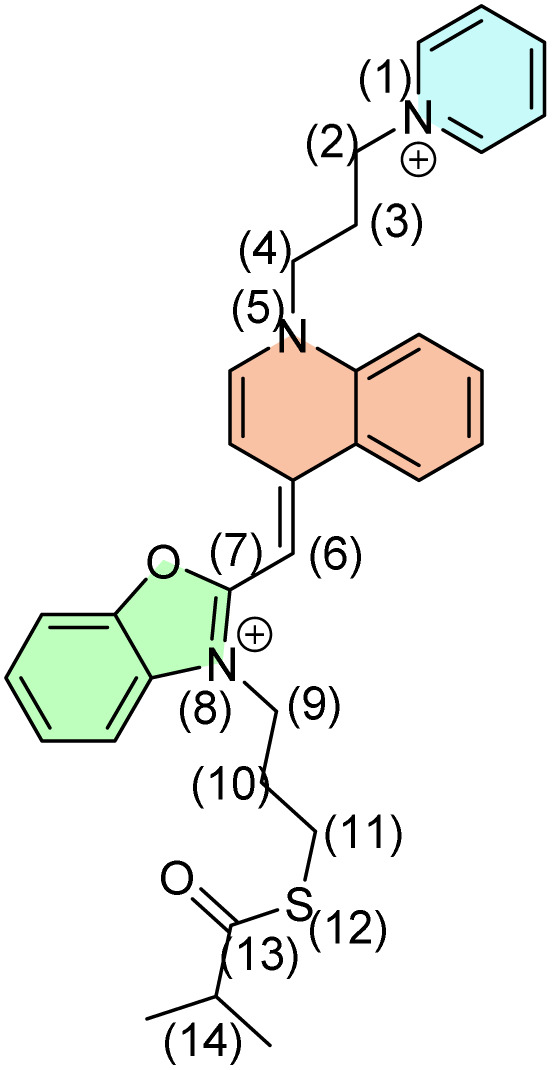	N1–C2	35.8	Heterolytic (carbocation C2^+^ and pyridine)
	C2–C3	30.6	Homolytic
	C3–C4	26.9	Homolytic
	C4–N5	22.0	Homolytic
	C6–C7	141.8	Homolytic
	N8–C9	59.0	Heterolytic (carbocation C9^+^ and benzoxazole)
	C9–C10	76.3	Homolytic
	C10–C11	71.7	Heterolytic (carbocation C11^+^ and carbanion C10^−^)
	C11–S12	63.5	Homolytic
	S12–C13	65.4	Homolytic
	C13–C14	78.3	Homolytic

**Scheme 3:**
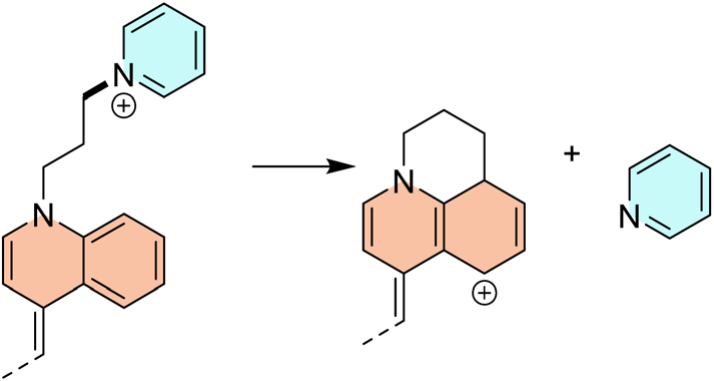
Carbocation rearrangement following the heterolytic C–N cleavage that liberates neutral pyridine.

Also, the heterolytic N8–C9 cleavage liberates neutral benzoxazole, requiring 59.0 kcal mol^−1^, and surpassing the homolytic analogue by 14.3 kcal mol^−1^. Particularly interesting in the equivalent process at the N1–C2 bond that offers a neutral pyridine and the C2-centered carbocation. The matching heterolytic bond dissociation energy of 35.8 kcal mol^−1^ appears rather low, and it occurs because of the subsequent carbocation rearrangement with the additional C–C bond formation (Scheme 3).

Because of our particular interest in this fragmentation pathway, we investigated pyridine liberation in more detail through kinetic aspects as well. Our computations showed this process is linked with a rather high kinetic barrier of Δ*G*^#^ = 70.3 kcal mol^−1^. The latter renders this bond extremely stable under normal conditions, yet likely cleavable under MS measurements. This is further confirmed by analogously analyzing a thermodynamically much more favorable C4–N5 cleavage with a bond dissociation energy of only 22.0 kcal mol^−1^. There, the computed kinetic barrier is even higher at Δ*G*^#^ = 71.6 kcal mol^−1^, thus justifying the sufficiency of thermodynamic considerations alone.

For halide complexes, our results show that they are able to exert their anion–π interaction tendency by preferably binding system 2.1 in the “sandwich”-type complexes through positioning between the pyridine and nitrogen-containing heterocycle (Fig. 9). Still, in line with earlier literature reports,^17^ the binding affinity for Cl^−^ is highest and assumes Δ*G*_BIND_ = −157.2 kcal mol^−1^, while for Br^−^ and I^−^ it is lower at −144.3 and −133.1 kcal mol^−1^, respectively. Yet, all these values are highly exergonic, thereby indicating their stability in the gas phase and signifying the preference of investigated dyes for halide complexes. Interestingly, the conformation of the complex that was most preferred for Cl^−^ in solution is 4.1 kcal mol^−1^ less stable in the gas-phase, being even higher at 5.1 kcal mol^−1^ for Br^−^ and 7.3 kcal mol^−1^ for I^−^. However, the fact that for all halides the dominant complexation involves their positioning between two aromatic groups, implies that their effect on the fragmentation patterns involving or close to those two moieties will be the largest.

**Scheme 4:**
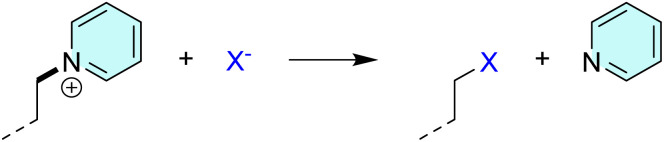
Schematic representation of the halide-assisted cleavage of the N1–C2 bond resembling S_N_2 process and liberating neutral pyridine.

In this context, let us focus on the N1–C2 fragmentation that releases neutral pyridine. Interestingly, it turns out that this process resembles a classical S_N_2 reaction mechanism, where the halide anion acts as a nucleophile and approaches the saturated C2-atom bearing pyridine (Scheme 4). In the identified transition state structures, the distances C–halide and C–N (pyridine) are 2.35 and 2.01 Å for Cl^−^, 2.53 and 2.03 Å from Br^−^, and 2.76 and 2.02 Å for I^−^, with the single negative vibration frequency of −480, −469, and −491 cm^−1^, in the same order. The reaction proceeds by liberating pyridine and forming of a new C2–halogen bond at the place of the cleavage (Scheme 4). This process is responsible for the existence of the peaks in the MS^*n*^ spectra appearing at 79 mass units lower than the molecular ion, the latter corresponding to the molecular mass of the released pyridine. However, the reaction is both kinetically and thermodynamically most favorable for iodide. Namely, for I^−^, the activation and reaction free energies are Δ*G*^#^ = 28.4 kcal mol^−1^ and Δ*G*_R_ = 3.0 kcal mol^−1^, thus outperforming Δ*G*^#^ = 29.2 kcal mol^−1^ and Δ*G*_R_ = 4.1 kcal mol^−1^ for Br^−^, and especially Δ*G*^#^ = 30.7 kcal mol^−1^ and Δ*G*_R_ = 6.0 kcal mol^−1^ for Cl^−^. Despite these differences, all three sets of values highlight a very large catalytic effect of halide anions, which assumes between 30–40 kcal mol^−1^ in both kinetic and thermodynamic aspects, being highly significant. Also, the thermodynamic difference of ΔΔ*G*_R_ = −3.0 kcal mol^−1^ in favor of I^−^ over Cl^−^, indicates around two orders of magnitude higher population of the iodine-containing fragments. The lower energetic cost of iodide-assisted cleavage is attributed to its higher polarizability and its ability to stabilize the developing charge in the transition state. This trend directly correlates with the MS^*n*^ experiments, which show lower collision energies required for fragmentation of iodide-containing complexes, enhanced retention of iodide in high-*m*/*z* product ions, and a greater abundance of iodide-stabilized fragments.

Furthermore, if we consider halogen derivatives formed upon the described N1–C2 cleavage (Scheme 4), they can undergo an 1,2-elimination of HX with the formation of the unsaturated C2=C3 bond according to the general scheme –CH_2_–CH_2_–X → –CH=CH_2_ + HX. Yet, this process is much more favorable for chlorine (Δ*G*^#^ = 62.6 kcal mol^−1^, Δ*G*_R_ = 16.6 kcal mol^−1^) than for iodine (Δ*G*^#^ = 63.1 kcal mol^−1^, Δ*G*_R_ = 21.3 kcal mol^−1^).

Lastly, given their preferred vicinity from the central aromatic core, halide anions can undergo an analogous S_N_2 nucleophilic reactivity towards the C4 atom, which releases the nitrogen-containing heterocycle and a saturated hydrocarbon with a new C4–halogen bond. However, the reaction parameters for this route suggest less favorable processes. Specifically, the computed activation energies are Δ*G*^#^ = 42.1 kcal mol^−1^ for Cl^−^ and Δ*G*^#^ = 38.8 kcal mol^−1^ for I^−^, while the reaction energies are Δ*G*_R_ = 29.1 kcal mol^−1^ for Cl^−^ and Δ*G*_R_ = 24.3 kcal mol^−1^ for I^−^. Although we again observe that both sets of data point to a higher reactivity of the iodine anion, these values suggest a lower importance of the C4–N5 fragmentation over the N1–C2 cleavage for both halogens, consistent with their limited experimental observation.

#### 3.4.3. Implications for halide-dependent MS behavior

Taken together, the computational results demonstrate that iodide plays a dual mechanistic role in the gas-phase behavior of cyanine dyes. While not the strongest halide binder in solution, iodide forms flexible and highly reactive gas-phase complexes that promote both intermolecular self-assembly and low-energy, selective fragmentation. The combination of anion–π interactions and S_N_2-like halide-assisted C–N bond cleavage provides a unified explanation for the experimentally observed iodide-driven clustering, halide-dependent fragmentation energetics, and distinctive MS^*n*^ patterns.

These findings support the conclusion that halide ions (particularly iodide) should be regarded as active participants rather than passive counterions in the gas-phase chemistry of multiply charged cyanine dyes.

## 4. Conclusion

In this work, we combined high-resolution tandem mass spectrometry and density functional theory calculations to elucidate the halide-dependent gas-phase behavior of asymmetric cyanine dyes across multiple charge states. The results demonstrate that halide counterions play an active mechanistic role, rather than serving merely as charge-balancing species. Structural characterization revealed that the main fragmentation pathways involve loss of halide counterions (I^−^, Br^−^, Cl^−^). Tandem MS and computational analysis confirmed iodide positioning between the benzene and DABCO moieties, with key CID fragments at *m*/*z* 427 (iodide bound to benzene) and *m*/*z* 253 (iodide bound to DABCO).

Computational studies, using complex 2.1 as an example, demonstrated feasible formation of both [2.1–Cl^−^]^+^ and [2.1–I^−^]^+^ in aqueous solution. While [2.1–Cl^−^]^+^ formation is highly exergonic, the [2.1–I^−^]^+^ complex retains sufficient stability for around 15% solution population, accounting for its observed biological activity and characteristic gas-phase fragmentation. Halide anions most strongly influence C–N bonds linking the propyl chain to pyridine or other nitrogen-containing aromatic units. Although homolytic cleavage is generally favored across various C–C and C–heteroatom bonds, both halides promote heterolytic cleavage *via* S_N_2 nucleophilic attack. These pathways are kinetically and thermodynamically preferred for I^−^ over Cl^−^, explaining their dominance in MS^*n*^ spectra. Such reactions release aromatic units and form new C–halogen bonds. There, chlorine facilitates slightly more favorable 1,2-elimination, resulting in prominent HCl loss in [2.1–Cl^−^]^+^ spectra compared to minimal HI loss in the [2.1–I^−^]^+^ analog.

Overall, this study advances current understanding of cyanine dye mass spectrometry by directly linking counterion identity to gas-phase structure, stability, and fragmentation pathways. These findings will facilitate the interpretation of MS data for cyanine-labeled biomolecules and guide the design of functional dyes with tailored physicochemical and biological properties. Such advancements are particularly relevant given the widespread applications of cyanine dyes in biological imaging and sensing,^34^ cancer diagnostics,^35^ detection of circulating tumor cells,^36^ and lymph node imaging.^37–39^ Consequently, mass spectrometry emerges as a valuable tool for monitoring label stability and detecting modifications such as photobleaching or oxidation in clinical probes.

While gas-phase MS provides valuable insight into intrinsic anion–π interactions and fragmentation pathways, direct extrapolation to solution or biological environments must be made cautiously. In the absence of solvation and counterion screening, gas-phase structures may overemphasize electrostatic and dispersion contributions relative to solution-phase behavior, and thus cluster abundances cannot be directly interpreted as solution binding constants. Furthermore, although the chosen DFT approach is suitable for halogenated systems, the absolute energies of anion–π complexes should be regarded as qualitative. These findings should therefore be viewed as complementary to, but not substitutes for, solution-phase or biological measurements. Nevertheless, the observed halide-dependent trends align with our aqueous-phase DFT results and with previously reported biological behavior of the dyes, suggesting that gas-phase MS captures fundamental interaction preferences that may contribute to solution or cellular properties.

## Author contributions

This manuscript was written with the contributions of all authors. All authors have read and agreed to the published version of the manuscript.

## Conflicts of interest

The authors reported no conflict of interest.

## Supplementary Material

RA-016-D5RA07246H-s001

## Data Availability

Mass spectrometry and computational data will be deposited in the Ruder Boskovic Institute public repository Fulir. Supplementary information: additional mass spectrometry spectra and structural conformations of the dyes used in the study. See DOI: https://doi.org/10.1039/d5ra07246h.
