# Supplementary material for: Combined mass spectrometry and computational analysis of cyanine dyes and their structural analogues
Source: RSC Adv. 2026 Jan 28;16(7):5770–84. doi: 10.1039/d5ra07246h (PMC12848875; doi:10.1039/d5ra07246h)
Supplement: RA-016-D5RA07246H-s001 [file RA-016-D5RA07246H-s001.pdf]

Supplementary Information (SI) for RSC Advances.
This journal is © The Royal Society of Chemistry 2026

Supplementary material

Combined mass spectrometry and computational analysis of cyanine dyes and their structural analogues

Irena Đapić[1*], Renata Kobetić[2], Atanas Kurutos[3], Tana Tandarić[4], Ružica Šoić[2], Robert Vianello[4*]

[1]Laboratory for Synthetic Methodologies in Organic Chemistry, Division of Organic Chemistry and Biochemistry, Ruđer Bošković Institute, Bijenička cesta 54, 10 000 Zagreb, Croatia
[2]Laboratory for Biomolecular Interactions and Spectroscopy, Division of Organic Chemistry and Biochemistry, Ruđer Bošković Institute, Bijenička cesta 54, 10 000 Zagreb, Croatia
[3]Institute of Organic Chemistry with Centre of Phytochemistry, Bulgarian Academy of Sciences, Acad. G. Bonchev Str., Bl. 9, 1113 Sofia, Bulgaria
[4]Laboratory for the Computational Design and Synthesis of Functional Materials, Division of Organic Chemistry and Biochemistry, Ruđer Bošković Institute, Bijenička cesta 54, 10 000 Zagreb, Croatia

Corresponding authors:

Irena Dapic, idapic@irb.hr, Laboratory for Synthetic Methodologies in Organic Chemistry, Division of Organic Chemistry and Biochemistry, Ruđer Bošković Institute, Bijenička cesta 54, 10 000 Zagreb, Croatia

Robert Vianello, Robert.Vianello@irb.hr, Laboratory for the Computational Design and Synthesis of Functional Materials, Division of Organic Chemistry and Biochemistry, Ruđer Bošković Institute, Bijenička cesta 54, 10 000 Zagreb, Croatia

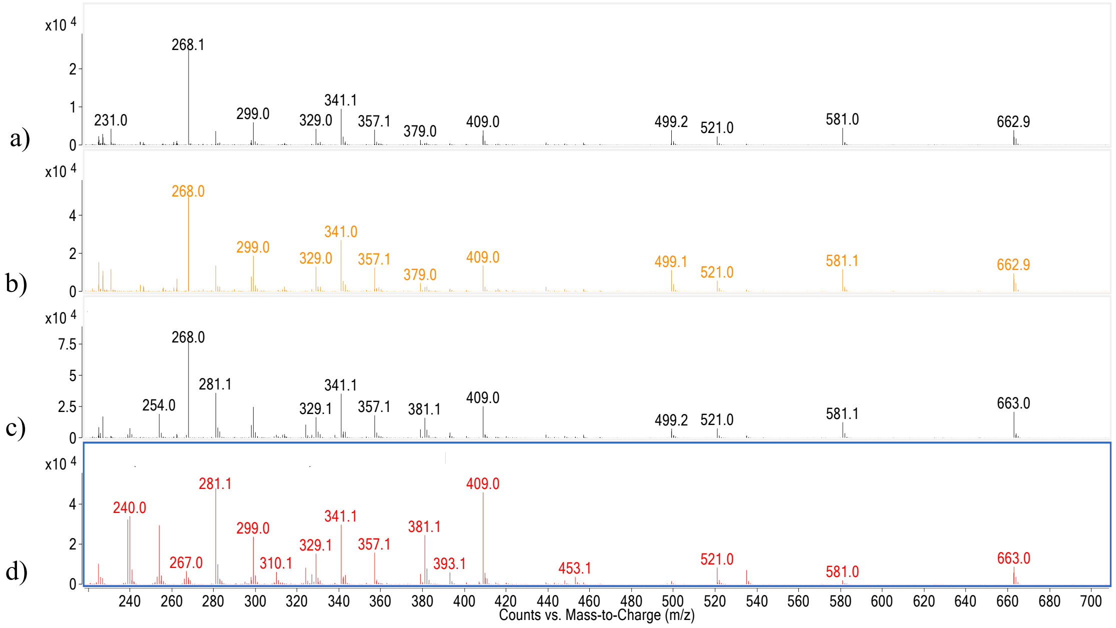

Supplementary figure 1. Comparison of different FV values and their effect on MS spectra of compound **3.2**. a) FV = 10 V; b) FV = 50 V; c) FV = 100 V; d) FV = 150 V. Data acquisition was performed on Agilent 6420 Triple Quad MS in methanol at concentration of 1 mg/mL.

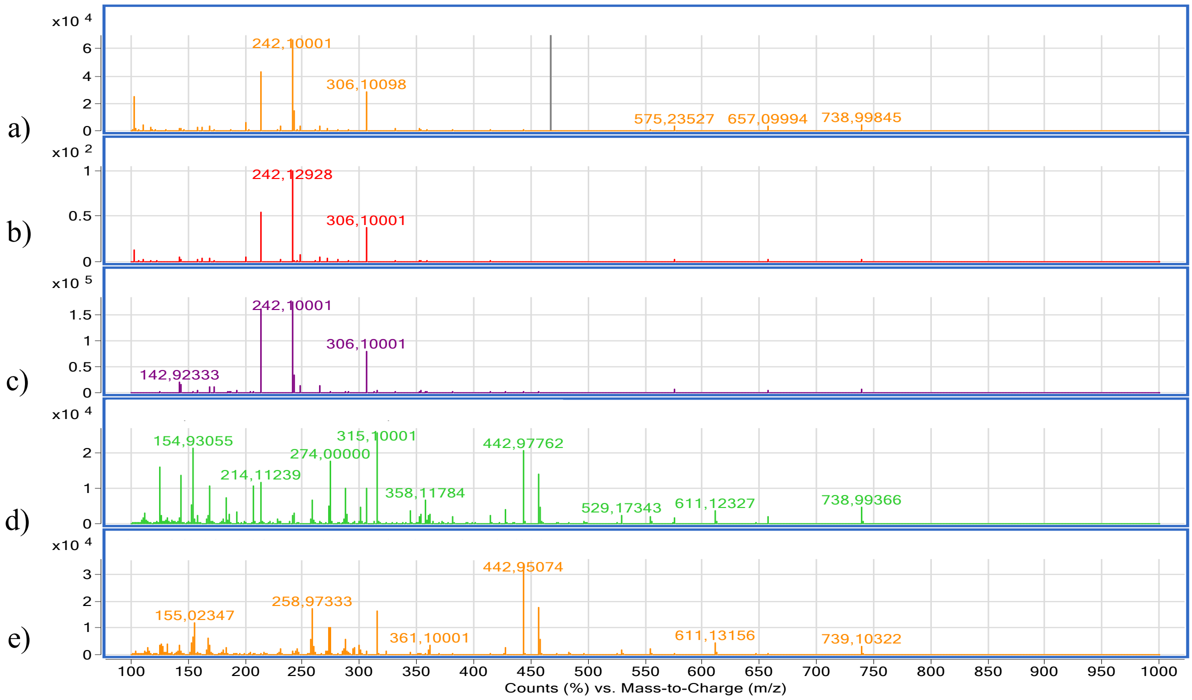

Supplementary figure 2. Comparison of different FV values and their effect on MS spectra of compound **3.1**. Increasing FV spectra show more rich MS spectra. a) FV = 10 V; b) FV = 50 V; C) FV = 100 V; d) FV = 150 V, e) FV = 200 V. Data acquisition was performed on Agilent 6420 Triple Quad MS in methanol at concentration of 1 mg/mL.

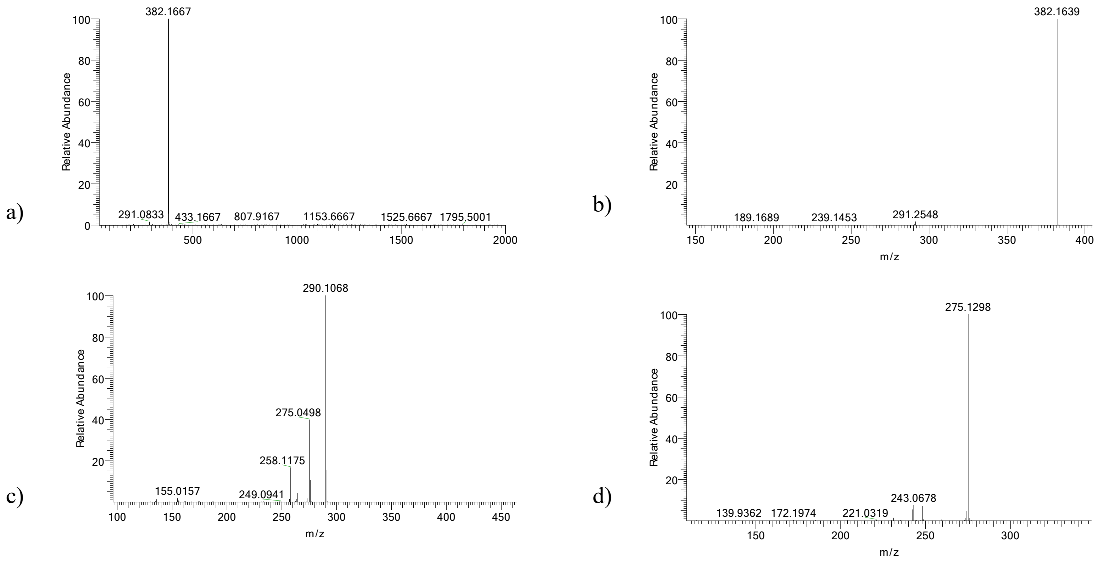

Supplementary figure 3. a) TIC mass spectra of compound **1**; b) MS^2 spectra of *m/z* 382, CID at 20 eV; c) MS^4 spectra of *m/z* 382 at CID 35 eV→291 at CID 35 eV→258 CID 40 eV; d) MS^4 spectra of *m/z* 382 at CID 35 eV→291 at CID 35 eV→275 at CID 40 eV. Data acquisition was performed on Orbitrap XL in water:methanol (1:1) at concentration of 1 mg/mL.

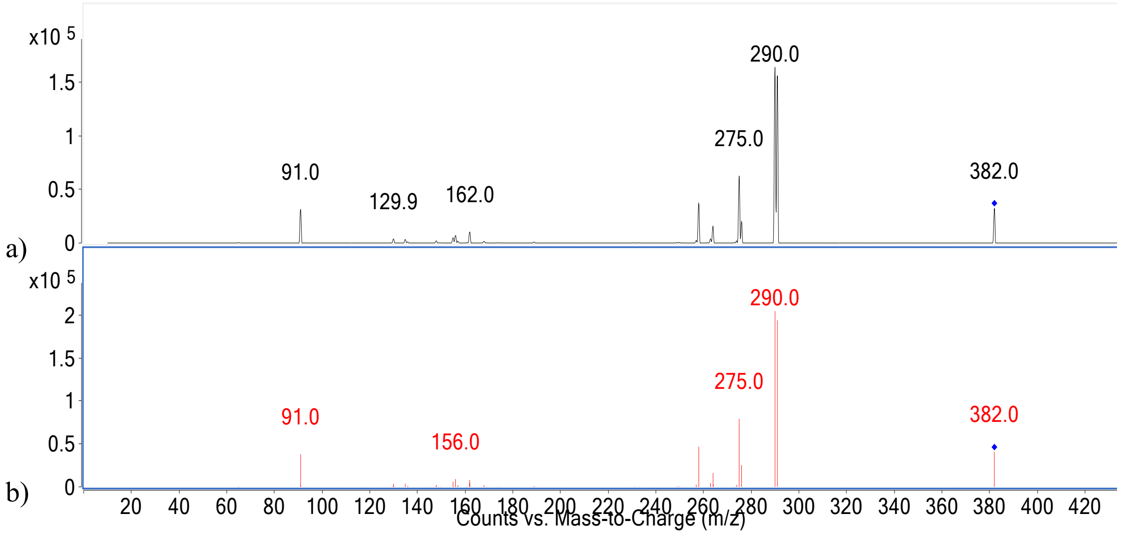

Supplementary figure 4. Product ion spectra for *m/z* 382 of compound **1** at CID 30 eV. a) FV = 50 V; b) FV = 100 V. Data acquisition was performed on Agilent 6420 Triple Quad MS in methanol at concentration of 1 mg/mL.

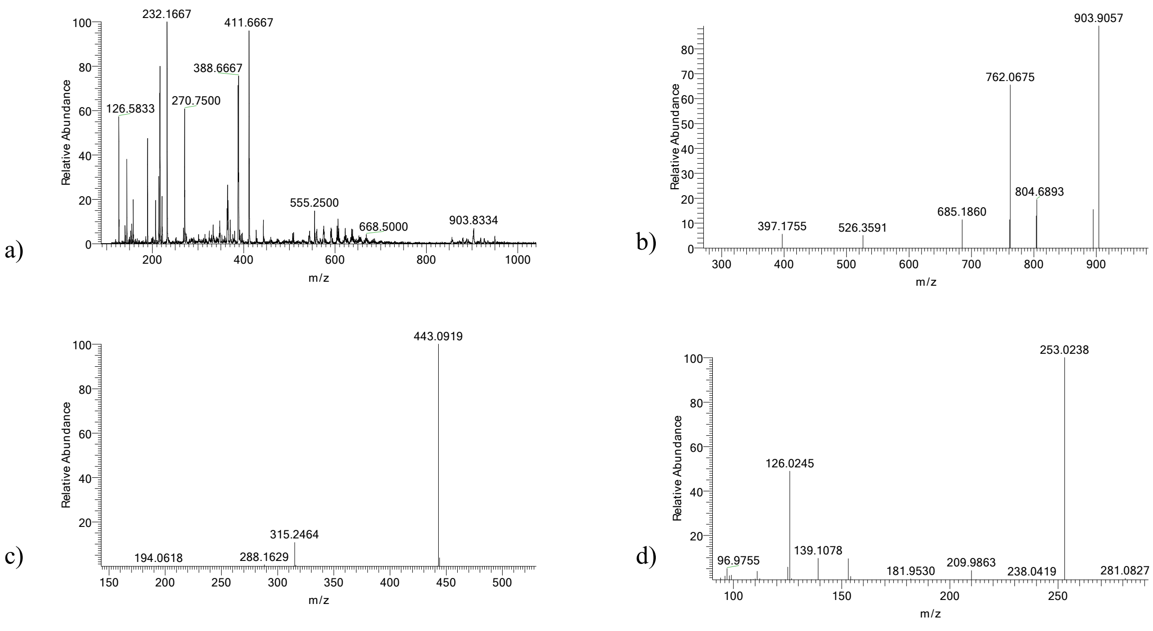

Supplementary figure 5. a) TIC mass spectra of compound **4**; b) MS^2 spectra of *m/z* 903 at CID 20 eV; c) MS4 spectra of *m/z* 412 at CID20 eV→ 555 at CID 30 eV→443 at CID20; d) MS4 spectra of *m/z* 412 at CID20 eV→555 at CID 30 eV→281 at CID30. Data acquisition was performed on Orbitrap XL in water: methanol (1:1) at concentration of 1 mg/mL.

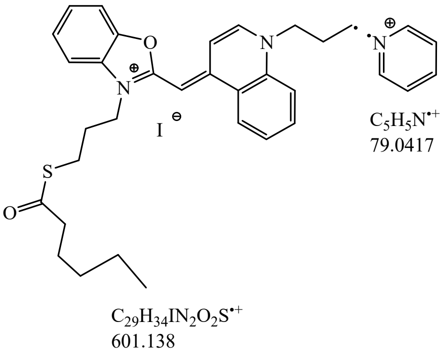

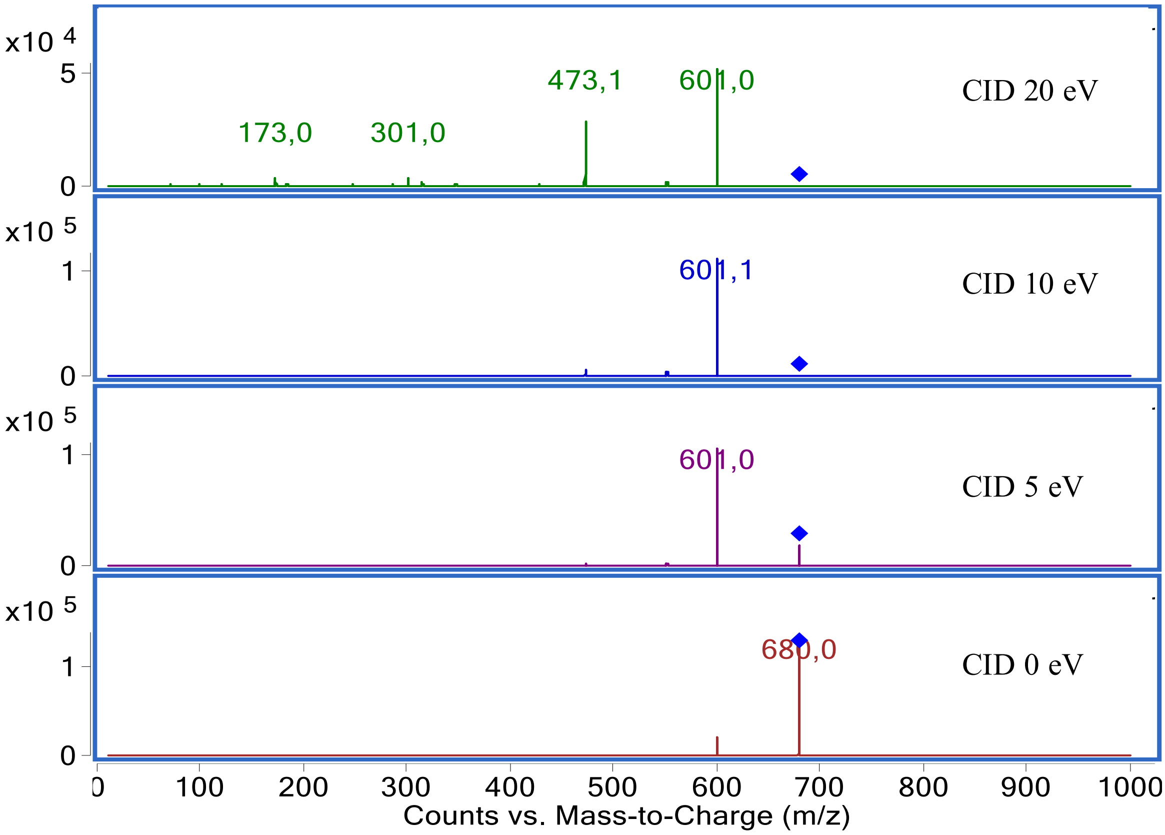

Supplementary figure 6. Product ion spectra of the detected anion-π complex of compound **2.5** (*m/z* 680.0) having I^- as counter ions. The fragmentation of the anion-π complex cyanine dye iodide. The CID experiments at the cone voltage 135V were performed for **2.5** dissolved in water:MeOH (1:1) at concentration of 10^{-5} mol/L. Data acquisition was performed on Agilent 6420 Triple Quad MS.

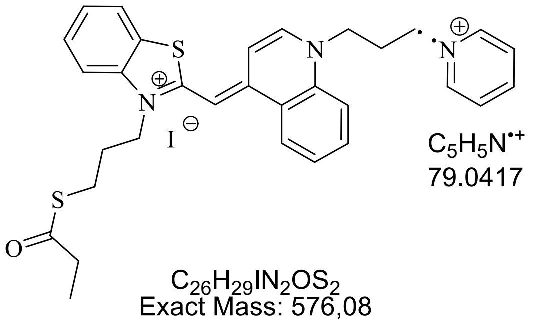

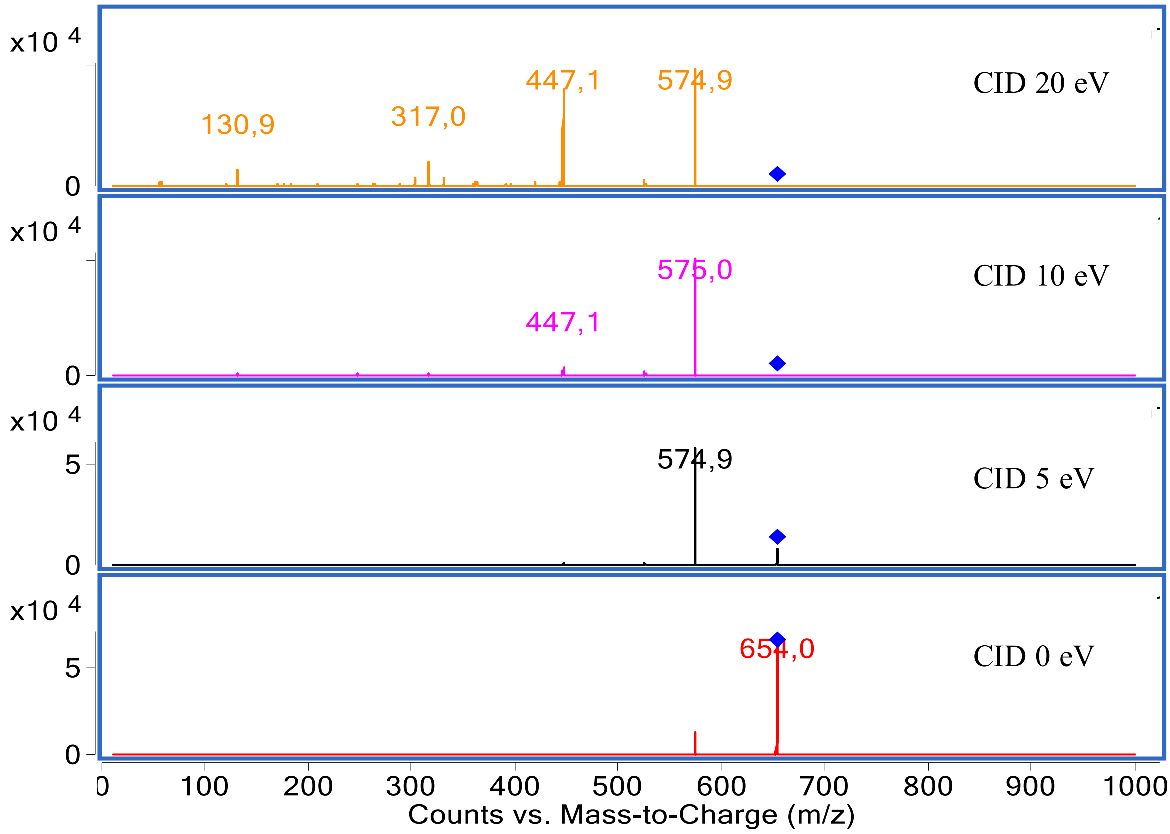

Supplementary figure 7. Product ion spectra of the detected anion-π complex of compound **2.4** having I^- (*m/z* 654.0) as counter ions. The fragmentation of the anion-π complex cyanine dye iodide. The CID experiments at the cone voltage 135V were performed for compound **2.4** dissolved in water:MeOH (1:1) at concentration of 10^{-5} mol/L. Data acquisition was performed on Agilent 6420 Triple Quad MS.

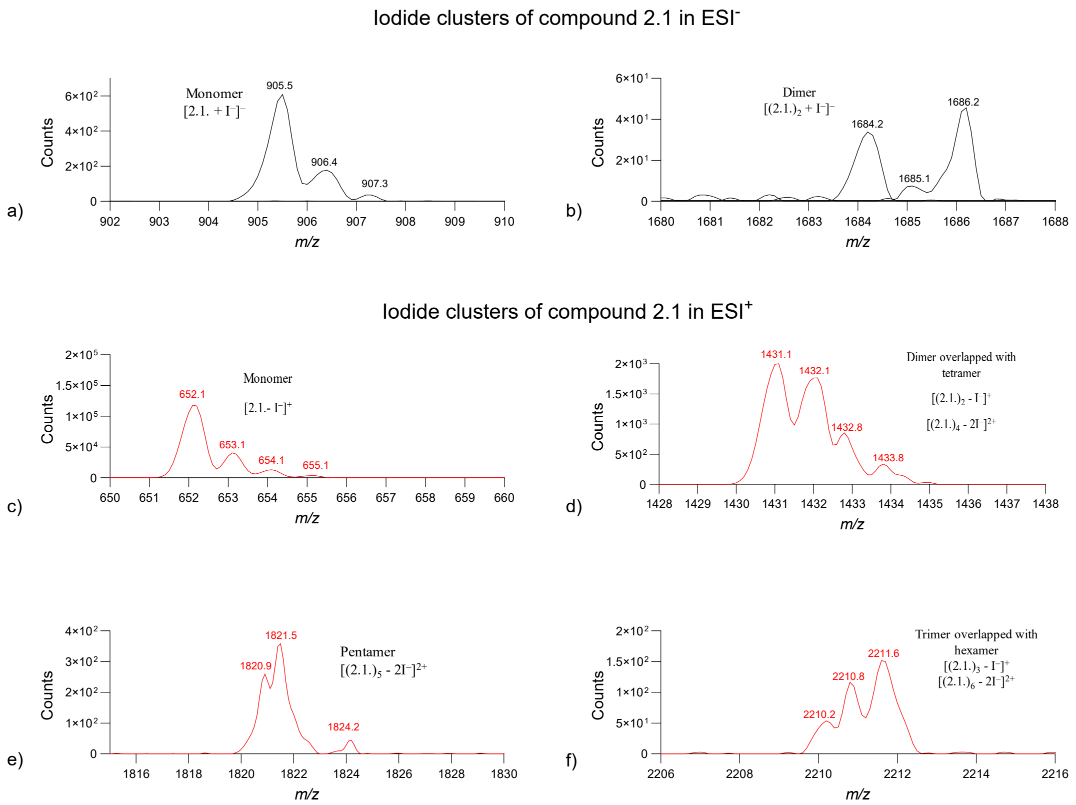

Supplementary figure 8. Iodide-driven self-assembly of cyanine dye **2.1** into higher-order gas-phase clusters (1:1 water:methanol used a solvent). Up to six compounds were detected in the cluster due to noncovalent interactions in ESI$^+$; a) MS ESI$^–$ spectra of monomer at *m/z* 905; b) MS ESI$^–$ spectra of dimer at *m/z* 1684; c) MS ESI$^+$ spectra of monomer at *m/z* 652; d) MS ESI$^+$ spectra dimer overlapped with tetramer; e) MS ESI$^+$ spectra of pentamer at *m/z* 1821; c) MS ESI$^+$ spectra of trimer overlapped with hexamer.

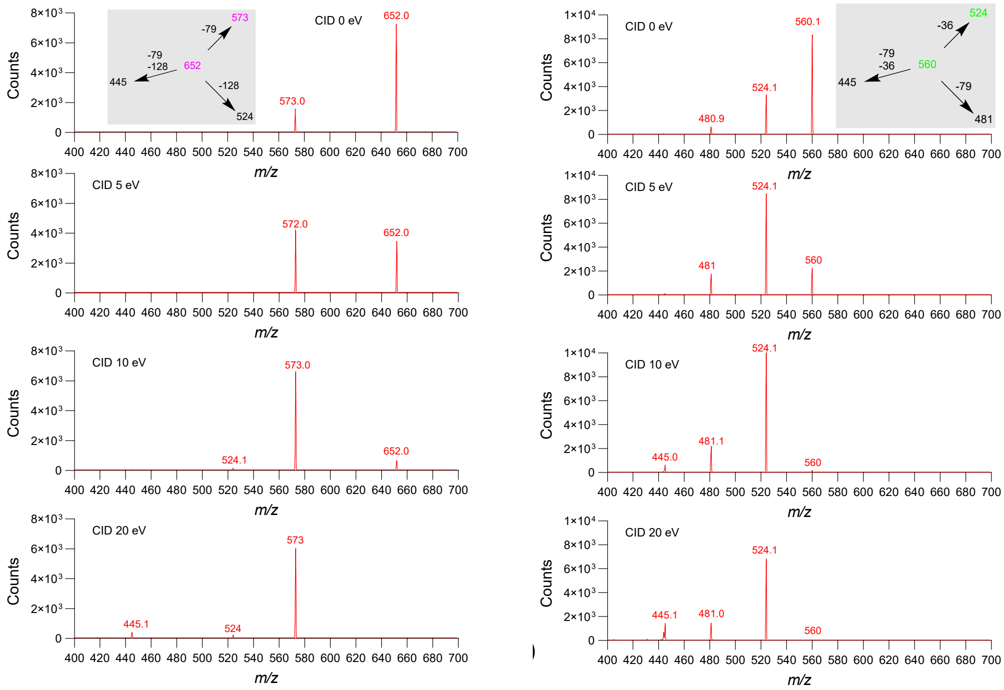

Supplementary figure 9. Halide-dependent MS/MS fragmentation energetics for iodide- (a, at *m/z* 652) and chloride-bound (b, at *m/z* 560) cyanine dye **2.1**. Iodide-containing complexes fragment at lower collision energies and retain the halide in key product ions, consistent with enhanced gas-phase stabilization. Data acquisition on Agilent 6420 Triple Quad MS was performed at CID of 0, 5, 10 and 20 eV to annotate molecular structures given as insets.

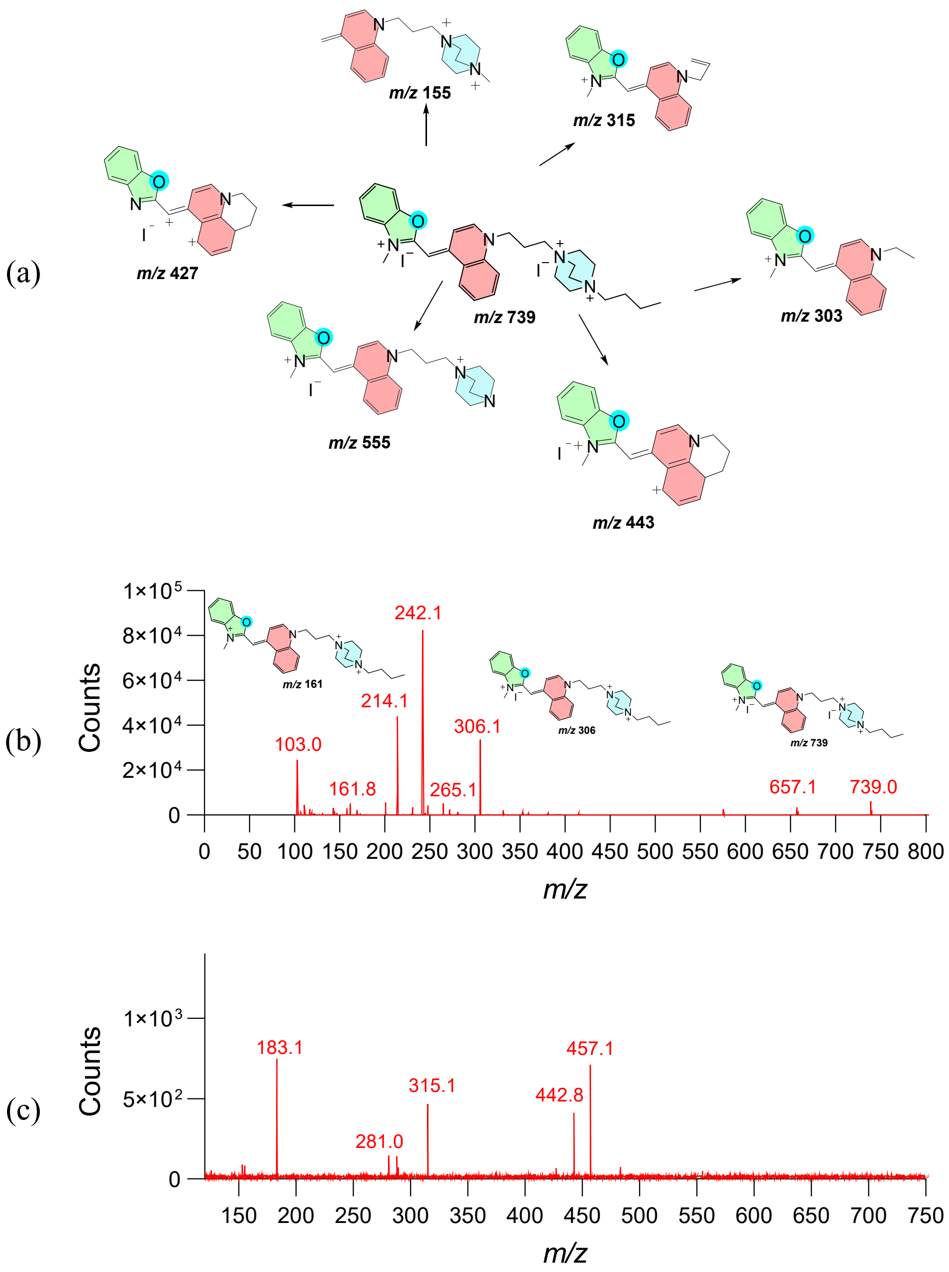

Supplementary figure 10. Representative Ms and MS/MS spectra of a tricationic cyanine dye **3.1** acquired on Agilent 6420 Triple Quad MS in methanol (c = 1 mg mL^{-1}). Fragmentation pathways closely mirror those of compound **4**, confirming the generality of halide-dependent behavior across charge states. a) Annotated molecular structures and fragmentation scheme of parent ion (*m/z* 739) assigned as $[M–I^{-}]^{+}$; b) TIC mass spectra and annotated structures of molecular ions (*m/z* 161, 306, and 739) assigned as $[M–3I^{-}]^{3+}$, $[M–2I^{-}]^{2+}$ and $[M–I^{-}]^{+}$, respectively; c) MS^2 spectra of *m/z* 739 at CID 40 eV.

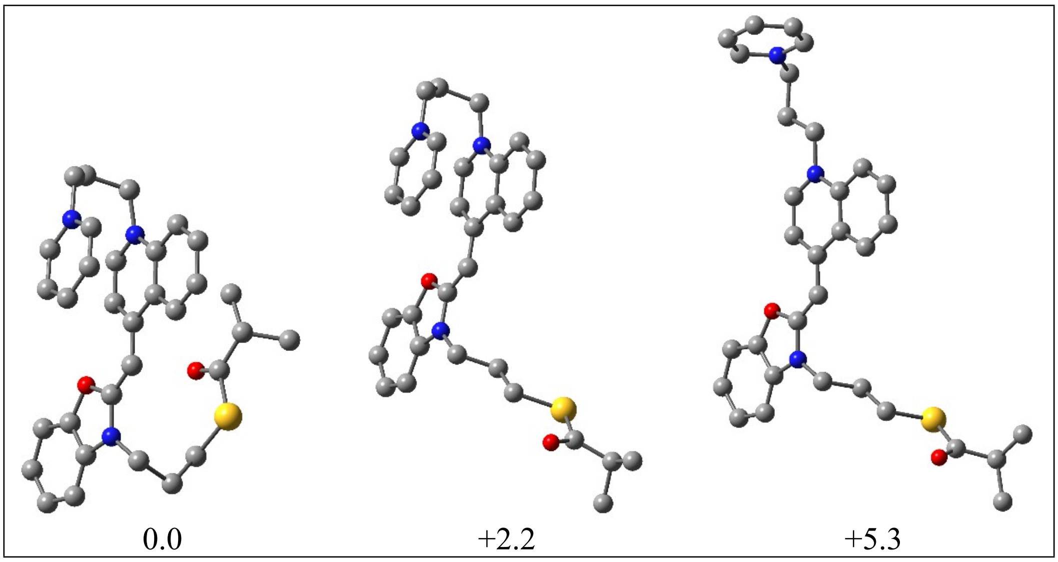

Supplementary figure 11. Relevant conformations of system **2.1** in the aqueous solution and their relative stabilities (in kcal mol^{-1}) as obtained at the (SMD)/M06–2X/LANL2DZdp level of theory following the CREST analysis. Hydrogen atoms are omitted for clarity.

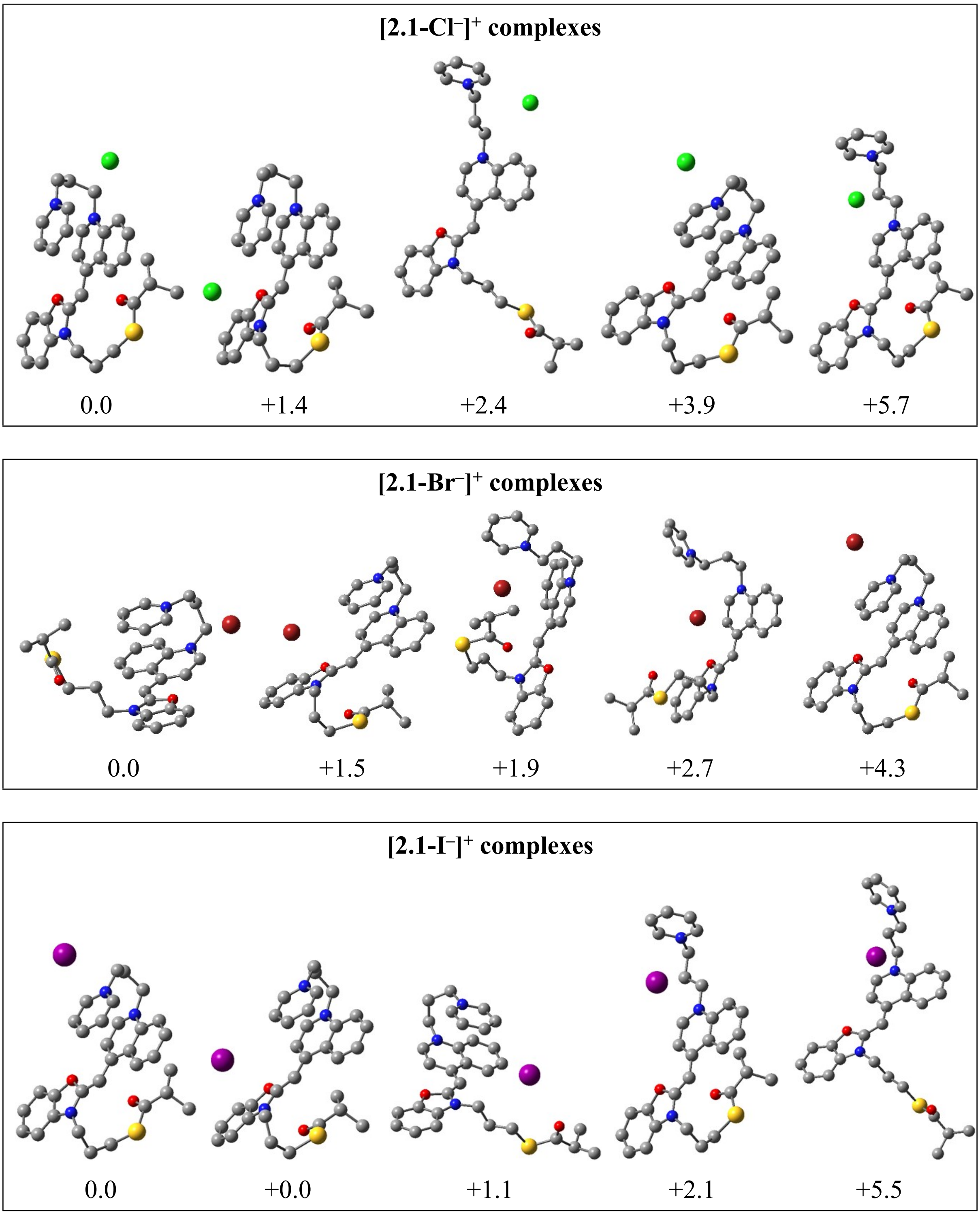

Supplementary figure 12. Relevant aqueous-phase conformations of complexes involving system **2.1** with halogenide anions, Cl^- (top), Br^- (middle) or I^- (bottom) and their relative stabilities (in kcal mol^{-1}) as obtained by the (SMD)/M06–2X/LANL2DZdp model following the CREST analysis. Hydrogen atoms are omitted for clarity.
